# MiR-146b-3p regulates proliferation of pancreatic cancer cells with stem cell-like properties by targeting MAP3K10

**DOI:** 10.7150/jca.48418

**Published:** 2021-05-03

**Authors:** Min Zhou, Yang Gao, Min Wang, Xingjun Guo, Xu Li, Feng Zhu, Simiao Xu, Renyi Qin

**Affiliations:** 1 Department of Biliary-Pancreatic Surgery, Affiliated Tongji Hospital, Tongji Medical College, Huazhong University of Science and Technology, Wuhan, 430030, China.; 2 Department of Endocrinology, Affiliated Tongji Hospital, Tongji Medical College, Huazhong University of Science and Technology, 430030, Wuhan, China.

**Keywords:** pancreatic cancer, stem cell, gene expression profiling, miR-146b-3p, MAP3K10

## Abstract

**Purpose:** Cancer stem cells (CSCs) initiate and maintain tumorigenesis due to their unique pluripotency. However, pancreatic stem cell gene signatures are not completely revealed yet. Here, we isolated pancreatic cancer stem cells (P-CSCs) and exploited their distinct genome-wide mRNA and miRNA expression profiles using microarrays.

**Methods:** CD24^+^ CD44^+^ ESA^+^ cells were isolated from two pancreatic xenograft cells by the flow cytometry and identified the stem cell-like properties by the tumor formation, self-renew and chemoresistance. Microarrays and qRT-PCR were used to exploit their distinct Genome-wide mRNA and miRNA expression profiles. The function and candidate target genes of key microRNA were detected after Ectopic restoration in the pancreatic cancer cell lines MIA Paca-2 (CSC^high^) and BxPC-3 (CSC^low^).

**Results:** In this study, we isolated P-CSCs from two xenografts cells. Genome-wide profiling experiments showed 479 genes and 15 microRNAs specifically expressed in the P-CSCs, including genes involved in TGF-β and p53 signaling pathways and particularly miR-146b-3p as the most significantly downregulated miRNA. We confirmed miR-146b-3p as a downregulated signature in pancreatic cancer tissues and cell line MIA Paca-2 (CSC^high^) cells. Ectopic restoration of miR-146b-3p expression with pre-miR reduced cell proliferation, induced apoptosis, increased G1 phase and reduced S phase in cell cycle in MIA Paca-2 (CSC^high^), but not in BxPC-3 (CSC^low^). Re-expression of miR-146b-3p with lentivirus significantly inhibited tumorigenicity *in vivo* in MIA Paca-2, but slightly in BxPC-3. Furthermore, we demonstrated that miR-146b-3p directly targeted MAP3K10 and might activate Hedgehog pathway as well through DYRK2 and GLI2.

**Conclusions:** These results suggest that P-CSCs have distinct gene expression profiles. MiR-146b-3p inhibits proliferation and induced apoptosis in P-CSCs high cells lines by targeting MAP3K10. Targeting P-CSCs specific genes may provide novel strategies for therapeutic purposes.

## Introduction

Pancreatic cancer is an aggressive malignancy with poor prognosis due to extensive local invasion, early systemic dissemination, and pronounced resistance to chemotherapy and radiotherapy 1. Accumulating evidences suggest that human pancreatic cancer possess cancer stem cells which contribute to tumor metastasis and therapy resistance 2. It has been reported that conventional chemotherapy is not capable of eliminating pancreatic cancer stem cells (P-CSCs), but rather enriches their numbers 3. It may explain why traditional chemotherapies initially shrink tumor size but fails to eradicate it, allowing eventual recurrence 4. Therefore, a better understanding of molecular mechanisms of P-CSCs is likely to lead to novel therapeutic strategies for pancreatic cancer.

MicroRNAs (miRNAs) are small noncoding regulatory RNA molecules, which profoundly affect a wide array of both normal and pathologic biological processes, including cancer 5, 6. Although increasing reports indicate that abnormal miRNA expression is connected to CSC dysregulation, only a few miRNAs, such as miR-34 have been studied for their roles in P-CSCs 7-9. The expression profiles and potential mechanisms of miRNAs in P-CSCs are still largely unknown. MiRNAs negatively regulate the expression of a wide variety of genes mainly through direct interaction with the 3'-untranslated regions (3'UTR) of their corresponding mRNA targets 10. In a broad array of developmental processes, miRNAs fine tune or restrict cellular properties by targeting important transcription factors or key pathways. Understanding of key target genes in P-CSCs is particularly important in miRNAs research. Recently, some key genes and pathways were identified in P-CSCs, such as Hedgehog (Hh), mTOR and NF-κB 11-13. It has also been reported that the combined blockade of sonic hedgehog and mTOR signaling together with standard chemotherapy is capable of eliminating P-CSCs. Though this evidence offered new potential treating strategies for pancreatic carcinoma, P-CSCs still could not be completely eliminated 14, 15. Therefore, further mechanism studies on key pathways and miRNAs are necessary and meaningful for more effective therapeutics against P-CSCs.

In this study, we isolated P-CSCs (CD24+CD44+ESA+) from two xenografts cells and exploited their distinct genome-wide mRNA and miRNA expression profiles using microarrays. We found a multitude of genes, involved in TGF-β and p53 signaling pathways and miR-146b-3p as the most significantly downregulated miRNA, differentially expressed in P-CSCs. We then demonstrated that miR-146b-3p regulated cell growth and tumorigenesis in MIA Paca-2 (CSC^high^), while not or slightly in BxPC-3 (CSC^low^) 14. Furthermore, we confirmed that miR-146b-3p directly targets MAP3K10, which may regulate the Hh signaling pathway. These results provide insights into the understanding of molecular mechanisms in P-CSCs regulation and may suggest a novel therapeutic strategy for pancreatic cancer.

## Materials and Methods

### Primary tumor samples and cell lines

Fresh and frozen human pancreatic cancer samples (n=12) and matched normal pancreatic tissues (n=12) were obtained from the Department of Surgery, Tongji Hospital Tongji Medical College, Huazhong University of Science and Technology, Wuhan China, during the period of time from Apr. 2016 to Oct. 2017 **(Supplemental Table 5)**. All human sample collection procedures were approved by the China Ethical Review Committee. Human pancreatic cancer cell lines Panc-1, SW1990, ASPC-1, PC-3, Mia-paca-2 and BxPC3 were obtained from Shanghai Cell Institute Country Cell Bank, (Shanghai China) and the Cell Resource Center of Xiang Ya Central Laboratory (Changsha, China). All the patients provided informed consent for sample collection and all samples were anonymized prior to the start of the study.

### Cell culture and transfection

All cell lines were grown in RPMI-1640 medium with 10% fetal bovine serum (FBS) (GIBCo/BRL, MD, USA), supplemented with 100 U/ml penicillin/streptomycin (Sigma). Cells were maintained at 37 °C in a humidified 5% CO_2_ incubator.

P-CSCs isolated from the xenograft were resuspended in DMEM-F12 without serum containing 20 ng/mL of epidermal growth factor (EGF), basic fibroblast growth factor (bFGF; both from PeproTech EC, London, UK), leukemia inhibitory factor (LIF; Chemicon), and B.27 (1:50; Life Technologies) as reported 4.

Cells were seeded in 6-well plates and transfected with miR-146b-3p precursor, or negative control (Ambion) at a final concentration of 50 nM using siPORT NeoFX Transfection Agent (Ambion) according to the manufacturer's protocol.

Empty vector (pGC FU-RNAi-NC-LV) and pGC miR-146b-3p-LV were constructed by and bought from SHANGHAI GENECHEM CO. Ltd. Cells were spin-infected with 1 mL of lentiviral supernatant containing 5 µg/mL polybrene for 2 hr at a multiplicity of infection of 1:10, followed by incubation for 2 hr at 37 °C. Transduction efficiency, evaluated by GFP expression, was >90%. Cells were harvested for further experiments 72 hr post-transfection.

### Cell sorting

The P-CSCs and non-P-CSCs were detected and sorted xenograft tumors according to the process previously described 2. All the pancreatic cells used to isolate CD24^+^ CD44^+^ ESA^+^ CSCs for further experiments were harvested from the first passage of the human tumor. The antibodies used were shown in **Supplemental Table 1**. Cells were routinely sorted twice, and the cells were reanalyzed for purity, which typically was > 90%.

### Microarrays

Total RNA was extracted and further purified using the Qiagen RNeasy Mini Kit and the Qiagen RNeasy Mini Column Kit (Valencia, CA, USA) according to manufacturer's instructions. MiRNA enrichment was performed with the mirVana miRNA Isolation Kit (Ambion, Austin, TX, USA) according to the manufacturer's instructions.

Genome-wide copy number analysis used Affymetrix U133 Plus 2.0 human oligonucleotide microarrays. Preparation of cRNA, hybridizations, washes, and detection were performed following the Affymetrix eukaryotic sample and array processing standard protocol.

MicroRNA microarray analysis used the Agilent Human miRNA Microarray Kit (V2) (Sanger database v.10.1). RNA labeling and hybridization on the Agilent miRNA microarray chips were performed with the miRNA Labeling Reagent and Hybridization Kit (Agilent Technologies, Santa Clara, CA, USA).

Genes were deemed significantly differentially expressed when P < 0.005, the false discovery rate (FDR) < 0.01, and an estimated absolute log 2-fold change > 0.5 in sequence counts across libraries.

Microarray Data Analysis Gene Ontology (GO) analysis, Pathway Analysis, Path-net, Signal-net, and MicroRNA-gene network were performed (Genminix Company, Shanghai, China) as previously described 16-19.

### Quantitative RT-PCR detection

Detection of the microRNAs was performed using Quantitect SYBR Green PCR Kit (Qiagen, Hilden, Germany) and quantitative RT-PCR Primer Sets (Ribobio, Guangzhou, China) with the U6 small nuclear RNA as an internal control. Data were analyzed according to the comparative Ct method. Detection of mRNA was performed using Quantitect SYBR Green PCR Kit (Qiagen), with GAPDH as an internal control. Sequences of primer pairs were shown in **Supplemental Table 2**. Amplification and detection were performed using CFX96 Real-Time PCR System (Bio-Rad).

### Cell proliferation, apoptosis, and cell cycle assay

After transfection with miR-146b-3p precursor or negative as described above, Cell proliferation, apoptosis, and cell cycle assay were analyzed by flow cytometry with a flow cytometer (FACScan, BD Biosciences).

Cell proliferation was evaluated at 24, 48 and 72 hours by using Carboxyfluorescein diacetate succinimidyl ester cell proliferation assay (CFSE; Invitrogen/Molecular Probes), according to the manufacturer's protocol.

The apoptosis assay was performed at 72 hours after transfection using the Annexin V-FITC Apoptosis Detection Kit I (BD Biosciences), according to the manufacturer's protocol.

The cell cycle assay was performed at 72 h after transfection. Cells were fixed in 70% ethanol at 4 °C for 16 h, stained with 50 mg/ml propidium iodide and 0.1 mg/ml RNase A.

### Xenograft tumorigenicity assay

The primary tumor xenografts were generated and treated using NOD/SCID mice according to methodology published elsewhere 2, 18. One xenograft was treated with gemcitabine 100 mg/kg twice weekly i.p for 8 weeks and passaged 3 times in the same way to detect the enrichment of CSCs. Xenograft tumors formed by subcutaneous injection of P-CSCs and non-P-CSCs isolated from xenograft tumors into mice, were processed as previously described.

Six-week-old male nude mice (BALB/c-nude) were used to examine tumorigenicity after transfection. Empty vector-transfected, and pcDNAmiR-146b-3p-transfected MIA PaCa-2 and BXPC-3 cells were propagated and 6 × 10^6^ cells were inoculated s.c. into the dorsal flanks of 5 mice respectively. For endpoint experiments, tumors were removed and weighed 7 weeks after tumor cell injection. All animal care was in accordance with the institutional guidelines. The animal study protocols were specifically approved by our institutional ethical review board.

### Plasmids construction and dual-luciferase reporter assay

We generated pGL3-MAP3K10 by amplifying a 282 bp 3'UTR fragment of MAP3K10 gene harboring the miR-146b-3p binding site predicted by the TargetScan *(http://www.targetscan.org/*, accession date Aug 20, 10) and subsequently cloning it into the pGL3-Promoter vector (Promega, Madison WI) at the XbaI site immediately downstream of firefly luciferase. The primer sequences used for amplification and the control vector pGL3-MAP3K10-mut, which has three mismatch mutations in the position 224-230 of MAP3K10 3'UTR (miR-146b-3p seed complementary sites) were shown in Supplemental Data 1. Luciferase activity was measured as described previously 7.

### Western blotting

Western blot analysis was carried out according to standard protocol and as described in**Supplementary Table 1.**

### Statistical analysis

All statistical analysis was done with SPSS16.0 software. Values are expressed as mean ± SD. Differences between groups were analyzed by Student's t-test or the nonlinear regression analysis between groups was used with logarithmic regression model by method of curve estimation. The level of statistical significance was set at P <0.05 for all tests.

## Results

### Isolation and identification of Human P-CSCs

**Figure 1:**
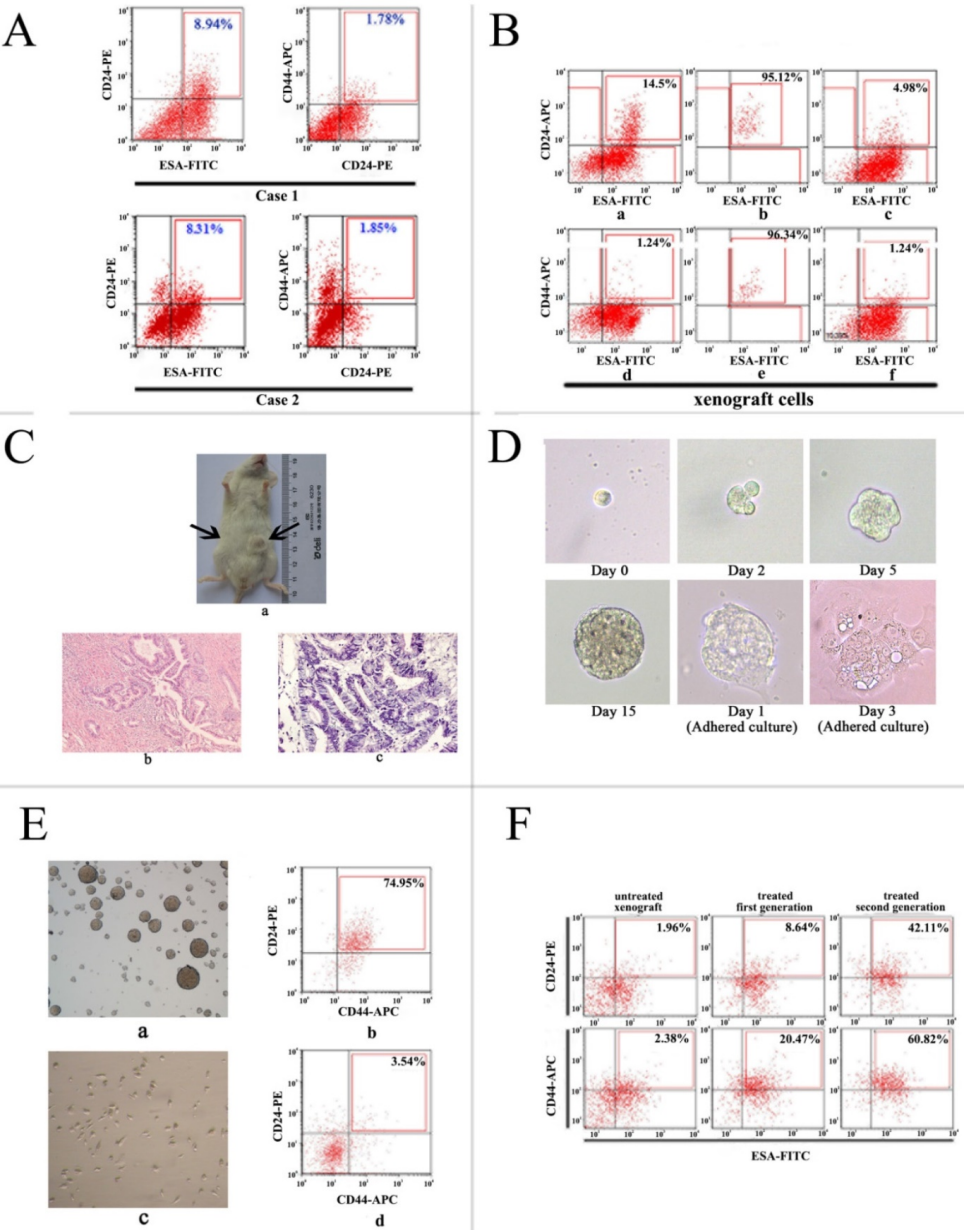
** Isolation and identification of P-CSCs. A)** Expression of CD24, CD44, and ESA in two pancreatic adenocarcinoma xenografts. **B)** CD24+ CD44+ ESA+ cells isolated xenograft cells show stem-cell-like properties. a and d: staining pattern in xenograft; b and e: purity analysis of isolated cells; C and F: staining pattern of the resultant tumor. **C)** Tumor formation in NOD/SCID mice**.** (a) a representative experiment depicting tumor formation in a mouse at the injection site of 1000 CD24+CD44+ESA+ cells, with no tumor formation seen at the injection site of 1000 CD24-CD44-ESAlow/- cells. H&E staining of the tumor generated from CD24+CD44+ESA+ cells (c) has similar histologic features to the corresponding patient's primary extrahepatic cholangiocarcinoma (b). Original magnification: b, c = ×400.**D)**Generation of a mammosphere from a single CD24+CD44+ESA+ grown in serum-free DMEM-F12 and adherent culture in DMEM supplemented with 10% FCS without growth factors. **E)** Detection of CD24+CD44+ cells in mammospheres and adherent cell of isolated cells**.** (a, b) nonadherent mammospheres cells; (c, d) adherent culture in DMEM supplemented with 10% FCS without growth factors.** F)** Increased percentage of CD24+ CD44+ ESA+ cells in the pancreatic cancer xenograft model after receiving gemcitabine and 5-FU treatment.

In the present study, we isolated and identified the P-CSCs by the markers of CD24, CD44 and ESA. In two pancreatic adenocarcinoma xenografts, the mean frequency of CD24^+^ CD44^+^ ESA^+^ cells were 1.2% and 0.8%, consistent with the report by Li et al. as shown in **Fig. 1A**. We purified CD24^+^ CD44^+^ ESA^+^ cells from xenograft cells **(Fig. 1B)**. These putative P-CSCs were used in subsequent experiments.

To validate CSC-like characteristics of isolated cells, tumor formation in immunodeficient mice, differentiation potential, spheroid-forming capacity, and enrichment of a CD44+/CD24+ /ESA+ phenotype by chemotherapy were detected. We found that positive xenograft cells (CD24^+^ CD44^+^ ESA^+^) could initiate tumors in 8/9 mice and negative xenograft cells (mainly CD24^-^, CD44^-,^ ESA^-/low^ cells) failed to form tumors in all mice injected by transplanting 10^3^ cells within 2 months** (Fig. 1C)**. Then the expression of phenotypes was analyzed in resultant tumors. The result showed that the highly tumorigenic cells produced additional positive cells, as well as phenotypically diverse non-tumorigenic cells showing the same phenotypic complexity as the primary tumor from which the tumorigenic cells were derived **(Fig. 1B)**.

To further test the self-renew properties of highly tumorigenic cancer cells *in vitro,* we cultured and isolated cells in serum-free medium supplemented with growth factors, within 2 to 3 weeks, pancreatic cancer spheres were observed in culture from primary P-CSCs **(Fig. 1D)**, while control cells were not. When isolated CSCs were grown as pancreatic spheres, a high percentage of the cells were CD24^+^ CD44^+^ ESA^+^ (mean > 60%). Under differentiating conditions (i.e., after withdrawal of growth factors and addition of 10% FBS), floating cells could adhere and differentiate. They acquired an epithelial-like morphology and returned to expressing low amounts of CD24^+^ CD44^+^ ESA^+^ cells (mean < 5%; **Fig. 1E)**.

According to the recent hypothesis that chemotherapy of tumors may lead to enrichment of CSCs, direct xenograft was used to test whether chemotherapy might enrich for P-CSCs. Of xenograft cells receiving gemcitabine treatment, 32% were CD24^+^ CD44^+^ ESA^+^ cells, compared with 0.8% of control pancreatic cancer cells receiving vehicle treatment **(Fig. 1F)**. These results suggested that a debulking approach could significantly enrich populations of precursor cells. All data suggested that the isolated cells from xenograft cells were pancreatic cancer cells, which retained tumorigenic activity and displayed stem cell-like properties.

### Genome-wide and microRNA expression profiling of P-CSCs

**Figure 2:**
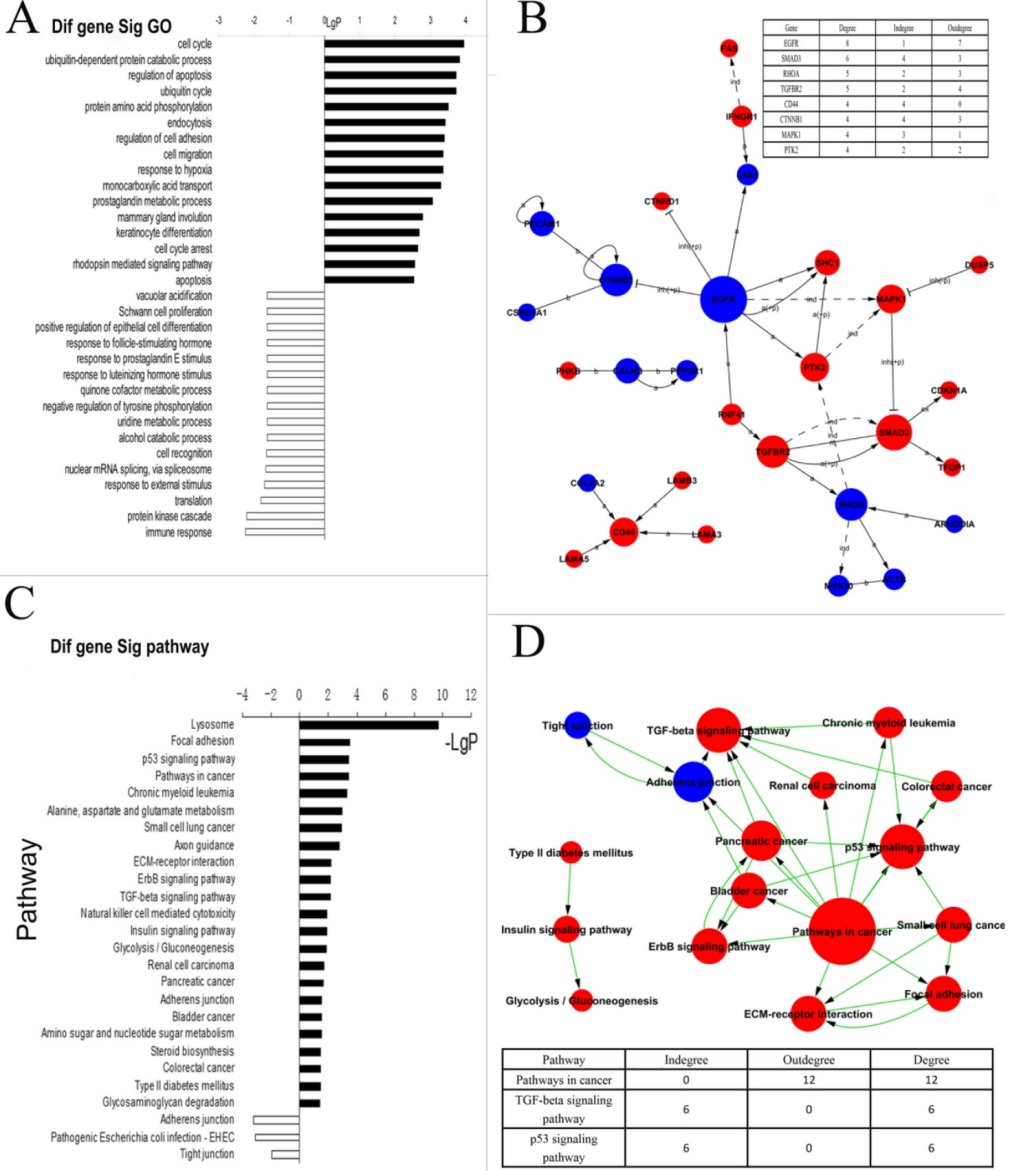
** Analysis of differentially expressed genes between Positive P-CSC cells and negative cells. A)** Biologic process-based gene ontology (GO) categories for differentially expressed genes. Main GO categories for upregulated genes are in black, on top; those for downregulated genes are white, on the bottom. **B)** KEGG pathway analysis for differentially expressed genes. The significant pathway for upregulated genes is in red; and in blue for downregulated genes. **C)** Signal network. The KEGG database was used to build a network of genes according to relationships among the genes, proteins and compounds in the database. A (active), inh (inhibit), p (phosphorylate), dp (dephosphorylate), ex (express), b (combined), and ind (indirect). The table on right top showed the key genes and the number of degrees. **D)** Path network. The KEGG database was used to construct a net of interactions of significant pathways of differentially expressed genes. The table on the down showed the key pathways and the number of degrees. upregulated genes are in red; and in blue for downregulated genes.

To determine differentially expressed genes, we firstly compared genome-wide expression patterns of P-CSCs derived from two xenograft cells. In total, we identified 341 up-regulated genes, including CD24 and CD44, and 138 down-regulated genes in CSCs from both primary cancers. The top 50 differentially up- and down-regulated genes are shown in **Table 1 (full list in Supplemental Table 3).** The 479 up- and down-regulated genes were analyzed by biological processes and molecular function, using information available from the Gene Ontology (GO) database. The annotated genes exhibited a wide range of functions. Genes involved in cell cycle, cell migration, apoptosis, cell growth, and immune response were shown to have potential roles in P-CSC development. The most statistically significant categories for CD44^+^ CD24^+^ ESA^+^ cells are shown in **Figure 2A (full list in Supplemental Table 4).** To further identify genes that play the most important roles in P-CSCs, the network of genes according to the relationships among the genes, proteins and compounds was built using the KEGG database. The data showed that *EGFR, SMAD3, RHOA, TGFBR2, CD44, CTNNB1, MAPK1* and *PTK2* were the most critical genes **(Fig. 2B)**.

To demonstrate whether these abnormal genes involved in some dysregulated signal pathways, we did some pathway analysis and found that ErbB-, p53-, and TGF-β signaling pathways, adherens junctions, etc. were abnormal in P-CSCs **(Fig. 2C)**. To further investigate interactions among the dysregulated pathways, and to determine the key pathways for CSC properties in the P-CSCs, the path-net was constructed according to the interactions among pathways shown in the KEGG database. As shown in **Fig. 2D**, signaling pathways for TGF-β and p53 appeared to play the most important roles in P-CSCs. The dysregulated genes in the TGF-β and p53 signaling pathway were showed in the **Supplemental Fig. 1**. Thus, global pathway analysis has identified critical biological networks disrupted in cancer compared with progeny cancer cells.

Because miRNAs regulate differentiation and can function as either tumor suppressors or oncogenes to regulate tumor development and prognosis, we examined whether different miRNA expression was observed between P-CSCs and more differentiated population. We studied miRNA expression profiles of P-CSCs derived from two xenograft cells using miRNA microarray. There were 128 and 138 miRNAs expressed differentially in P-CSCs, compared with their respective progeny cells. Analysis of all array data showed 15 genes with the similar alteration, including 7 up-regulated and 8 down-regulated miRNAs in CSCs compared with non-CSCs **(Table 1)**.

**Figure 3:**
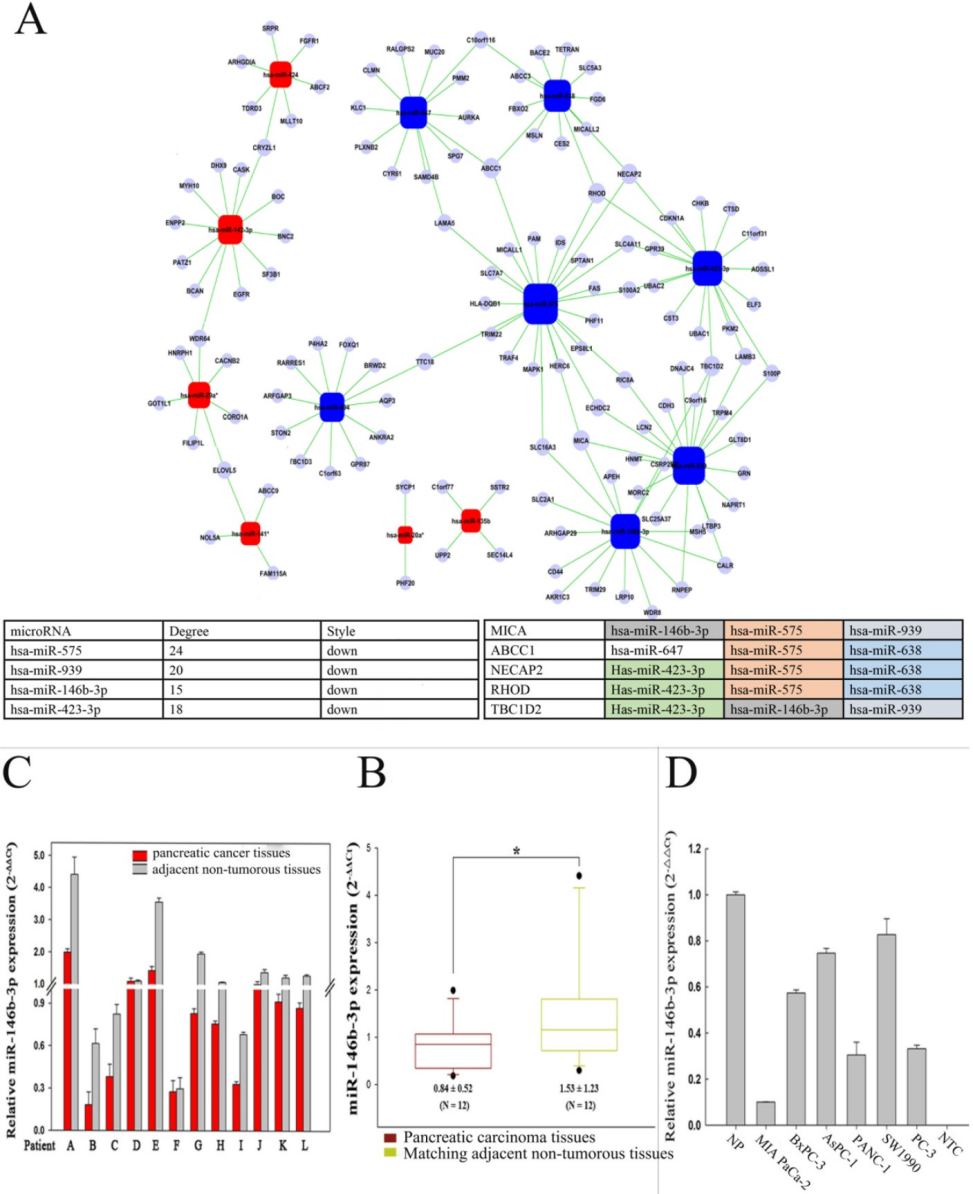
** Analysis of differentially microRNA between P-CSC cells and negative cells. A)** MicroRNA-gene network. Squares represent microRNA nodes; circles represent mRNA nodes. Edges describe inhibitive effects of microRNA on mRNA. Red box nodes show the over-expressed microRNA-mRNA network; blue box nodes show under-expressed microRNA-mRNA network. The table on down showed the key microRNAs and the key target genes detected aberrant by the gene-wild microarrays in P-CSCs. **B)** Relative miR-146b-3p expression levels in 12 pairs of pancreatic cancer tissues and matched normal pancreatic tissues by real time PCR. **C)** The data for miR-146b-3p expression was analyzed using the Mann-Whitney U test.** D)** Quantitative RT-PCR analysis of relative miR-146b-3p expression levels in human pancreatic cancer cell lines compared with normal pancreatic tissues (NP). Data were normalized to U6 control.

To further investigate target genes of the dysregulated miRNAs and determine key miRNAs in pancreatic CSCs, we analyzed the microRNA network **(Fig. 3A)**. The data showed that *hsa-miR-575*, *hsa-miR-939*, *hsa-miR-423-3p*, and *hsa-miR-146b-3p* were the most important dysregulated miRNAs. Furthermore, target genes of dysregulated miRNAs were predicted based on predicted base pairing using miRBase targets. The predicted target sequences were analyzed with the genome-wide expression patterns of P-CSCs. Based on the microRNA-gene network, several important target genes were determined, including *MICA*, *ABCC1*, *NECAP2*, *RHOD*, and *TBC1D2* **(Fig. 3A)**. Taken together, these results suggested that P-CSCs might have distinct Genome-wide and miRNA expression patterns that might regulate CSC properties.

### Expression and function of miR-146b-3p in pancreatic cancers and cells lines

Recent studies have demonstrated that the microRNA pathway is essential for controlling self-renewal, differentiation and transformation in normal and cancer stem cells. In order to determine crucial microRNAs in P-CSCs, we assessed the expressions of 15 differentially expressed microRNAs identified by microarray in 12 pairs of pancreatic cancer tissues and matched normal pancreatic tissues by RT-PCR. Our data showed that 6 microRNAs, including mir-146b-3p, decreased in pancreatic cancers **(Fig. 3B, C and Supplemental Fig. 2)**. Furthermore, analysis of 15 microRNAs expression in five pancreatic cancer cell lines revealed that different cells have different microRNA expression** (Supplemental Fig. 3)**. The expression of miR-146b-3p was significantly lower in Mia-paca-2 characterized as CSC^high^ than that in BxPC3 characterized as CSC^low^, with a median change of 5.74-fold **(Fig. 3D)**.

**Figure 4:**
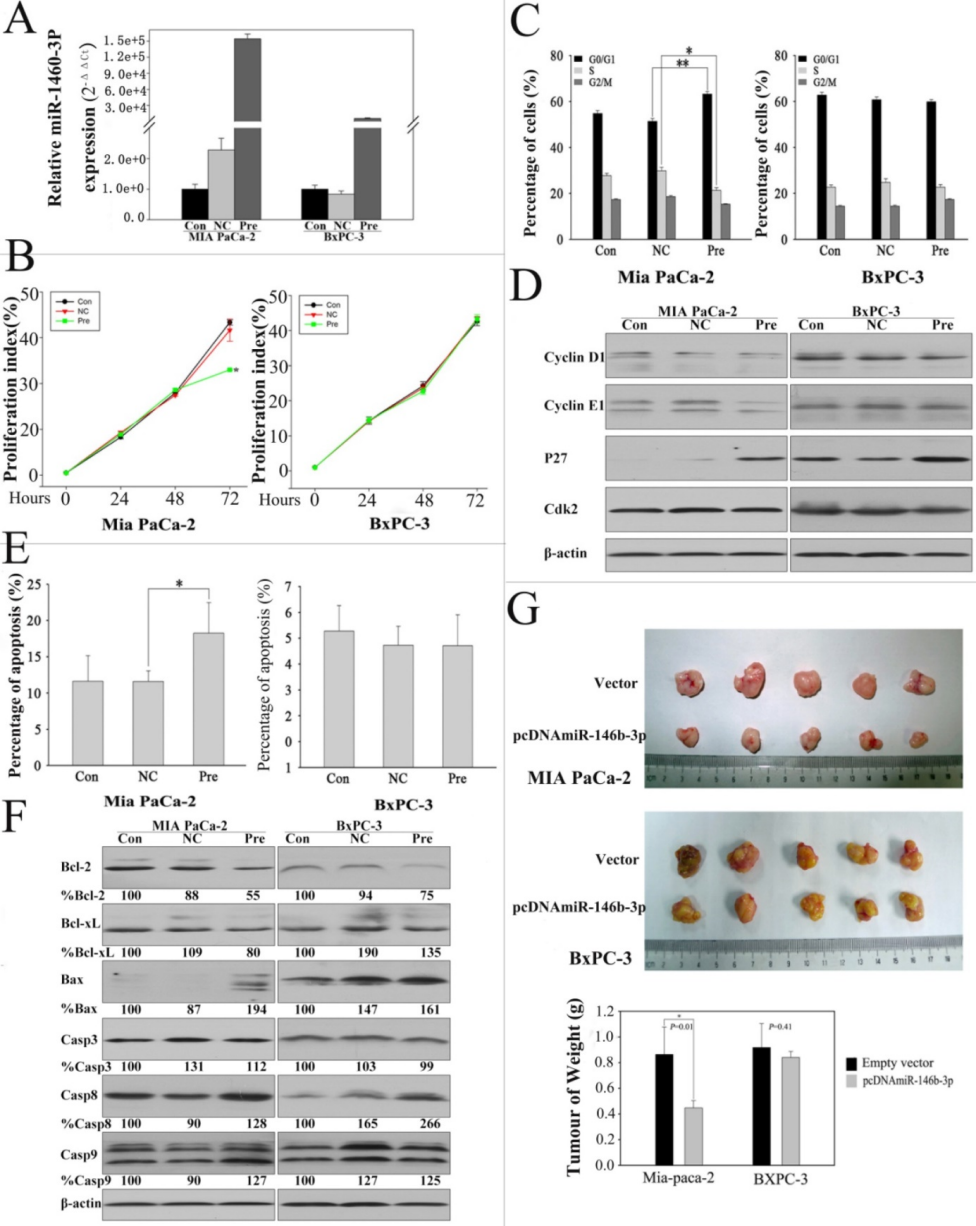
** Expression of miR-146b-3p after transfection with the miR-146b-3p and over-expression of miR-146b-3p affected cell growth, cell cycle, apoptosis, and tumorigenicity *in vivo*. A)** Expression of miR-146b-3p in miR-146b-3p mimic-transfected, mimic NC-transfected and un-transfected Mia PaCa-2 and BxPC-3 cells. **B)** Growth of MIA PaCa-2 and PANC-1 cells was shown after transfection with miR-146b-3p mimic or mimic NC or no transfection. The growth index was assessed at 1, 2, and 3 d by the CSFE assay. **C)** Over-expression of miR-146b-3p resulted in G1 arrest and S-phase increasing in Mia PaCa-2 and not in BxPC-3 cells as measured by PI staining. **D)** Cell cycle protein (Cyclin D, Cyclin E, P27, and Cdk2) were detected in MIA PaCa-2 and BxPC-3 cells by WB after transfection with miR-146b-3p mimic or mimic NC or no transfection. **E)** Apoptosis of MIA PaCa-2 and PANC-1 cells were detected by Annexin V/PI after treatment with miR-146b-3p mimic or mimic NC or no transfection in 72 h. **F)** Cell Apoptosis protein (Bcl-2, Bcl-xl, Bax, Casp3, Casp8, and Casp8) were detected in MIA PaCa-2 and BxPC-3 cells by WB after transfection with miR-146b-3p mimic or mimic NC or no transfection.** G)** Empty vector-transfected, and pcDNAmiR-146b-3p-transfected MIA PaCa-2 and BXPC-3 cells were propagated into nude mice. For endpoint experiments, tumors were removed and weighed 7 weeks after implantation. Tumor weight was obtained and presented. All data are shown as mean ± SD. *, P < 0.05; **, P < 0.01.

MiR-146b-3p was downregulated significantly and concordantly in P-CSCs detected by microarray and RT-PCR. In order to understand the function of miR-146b-3p in P-CSCs, we overexpressed miR-146b-3p in Mia-paca-2 and BxPC3 and analyzed for cell growth, cell cycle progression and apoptosis **(Fig. 4A)**. The CSFE proliferation assay showed that cell growth was reduced in pre-miR-146b-3p-transfected MIA PaCa-2 cells compared with scramble-transfected cells or untreated cells in 72h, but not in BXPC-3 cells **(Fig. 4B)**. The cell cycle analysis further confirmed this observation, indicating that pre-miR-146b-3p treatment induced cell cycle dysregulated in G1 phase with a significant increase in the percentage of cells in G1 phase (~14%) and a reduction of the S-phase cell population by ~23% in MIA PaCa-2, but no change in BXPC-3 **(Fig.4 C)**. To further confirm the change of the cell cycle in MIA PaCa-2, we detected critical factors of cell cycle by western blot. We found p27 protein upregulated in both cells lines, Cyclin E1 just upregulated in Mia-paca-2 (CSC^high^), Cyclin D1 and CDK2 were not changed after pre-miR-146b-3p transfection **(Fig. 4D)**. We also detected the apoptosis change in 72 hours after transfection, the data showed that pre-miR-146b-3p treatment induced apoptosis with a significant increase percentage (18.26%), compared with scramble-transfected cells (11.59%) or untreated cells (11.62%) in MIA PaCa-2, but no change in BXPC-3 **(Fig. 4E)**. Then we detected apoptosis-related proteins, and found Bcl-2 and Bcl-xL were downregulated, Caspase 8 was upregulated in both cell lines, Bax and Caspase 9 upregulated only in Mia-paca-2 (CSC^high^), Caspase 3 was not changed in both cell lines after pre-miR-146b-3p transfection **(Fig.4 F)**. Then we tested whether or not miR-146b-3p inhibited tumor growth *in vivo*. Empty vector-transfected and pcDNAmiR-146b-3p-transfected MIA PaCa-2 and BXPC-3 cells were injected s.c. into the posterior flank of nude mice. After 7 weeks, we found that tumor growth was significantly slower in the miR-146b-3p transfected mice than in the vector-treated in MIA PaCa-2 cells, but no change in BXPC-3 cells **(Fig. 4G)**.

### MAP3K10 is a direct target of miR-146b-3p

**Figure 5:**
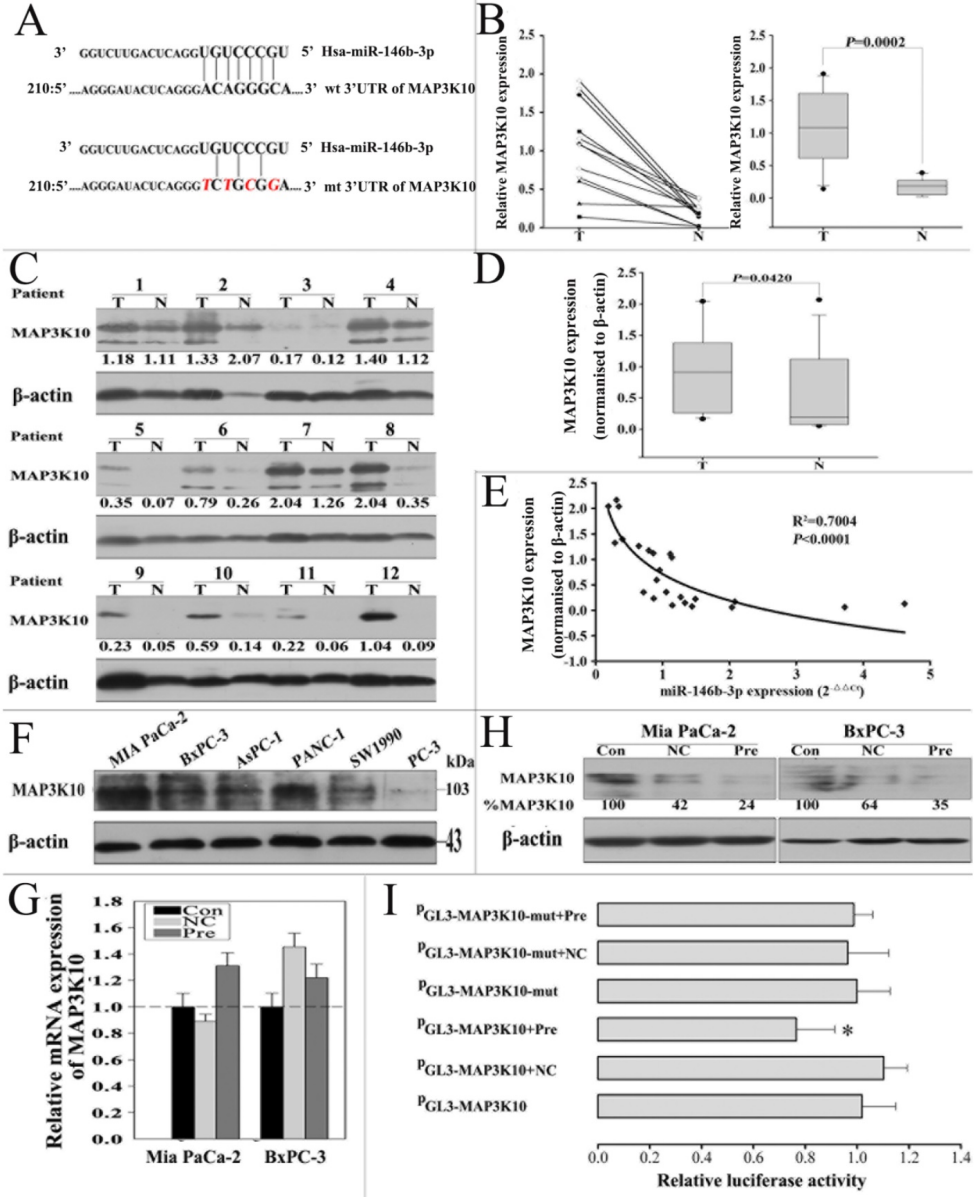
** miR-146b-3p negatively regulates MAP3K10 by binding to the MAP3K10 3'-UTR. A)** Sequence alignment of miR-146b-3p with the 3'-UTR of MAP3K10. The seed sequence of let-7a (top) matches the 3'-UTR of MAP3K10. Bottom, mutations of the 3'-UTR of MAP3K10 for creating the Renilla reporter constructs (the mutant nucleotides of the miR-146b-3p binding site are red and italic).**B), C), and D)**, The comparison of MAP3K10 expression between matched normal pancreatic tissue and pancreatic cancer tissues in 12 patients. (B) Analysis of the gene expression levels of MAP3K10 by real time PCR and (right) the data were analyzed using the Mann-Whitney U test. (C) Analysis of the protein expression levels of MAP3K10 by western blotting and (D) the data were analyzed using the Mann-Whitney U test. T, pancreatic cancer tumor tissue; N, pair-matched adjacent non-tumor tissue. **E)** Correlation of the protein expression of MAP3K10 with miR-146b-3p expression in 12 pairs of pancreatic cancer tissues and matched normal pancreatic tissues. Coefficient of determination from logarithmic regression model (R2) and P values are given (R2=0.7004; P<0.0001). **F)** Analysis of the protein expression levels of MAP3K10 in six pancreatic cancer cell lines by western blotting. **G)** Analysis of Gene expression of MAP3K10 in Mia PaCa-2 and BxPC-3 cells after transfection with miR-146b-3p mimic or mimic NC or no transfection by real time PCR.**H)** Analysis of protein expression of MAP3K10 in Mia PaCa-2 and BxPC-3 cells after transfection with miR-146b-3p mimic or mimic NC or no transfection by western blot.** I)** Analysis of luciferase activity. miR-146b-3p mimic inhibited wild type but not mutant MAP3K10 3'-UTR reporter activity. *P <0.05, compared to the mimic NC group. P values were obtained by two-tailed Student's t test.

To fully understand the mechanisms of miR-146b-3p, we adopted two bioinformatic algorithms (TargetScan, and miRanda) to identify a potential target gene. For its crucial role in Hedgehog signaling pathway confirmed as a key pathway in P-CSCs, MAP3K10 was selected for further analysis **(Fig. 5A)** 20. MAP3K10 expression in pancreatic cancer is still unclear, so we first examined MAP3K10 in the 12 matched normal and cancer tissues by RT-PCR and western blot. We found MAP3K10 can be detected in both cancer and matched normal tissues, but it was distinctly stronger in cancer tissues **(Fig. 5B, C and D)**. Then, we analyzed the correlation of the levels of the MAP3K10 protein with miR-146b-3p expression. There was a reversed nonlinear correlation between the level of the MAP3K10 protein and miR-146b-3p expression **(Fig. 5E)**. We also detected MAP3K10 expression in five pancreatic cancer cell lines and found MAP3K10 expression in all cell lines, but its level was significantly higher in Mia-paca-2(CSC^high^) than in BxPC3 (CSC^low^). Together, the expression of MAP3K10 was reversely associated with that of miR-146b-3p **(Fig. 5F)**.

To further test the hypothesis that MAP3K10 might be a target of miR-146b-3p, we overexpressed of miR-146b-3p by transecting with pre-miR-146b-3p or scramble. We found that the expression of MAP3K10 protein was downregulated in pre-miR-146b-3p-treated MIA-PaCa-2 cells, but not in scramble or untreated cells **(Fig. 5H)**. In addition, MAP3K10 mRNA expression was determined by real-time PCR. We observed no significant differences between pre-miR-146b-3p-treated and scramble-treated or untreated MIA PaCa-2cells **(Fig. 5G)**. To confirm that the MAP3K10 is a direct target of miR-146b-3p, a human miR-146b-3p 3'-UTR fragment containing a wild type or mutant miR-146b-3p binding sequence was cloned downstream of the luciferase reporter gene. Mia-paca-2 cells were co-transfected with reporter plasmid (MAP3K10-WT) and pre miR-146b-3p/scramble. As a result, pre-miR-146b-3p-transfected cells showed a marked reduction (≈77%) of luciferase activity, but the inhibition of luciferase activity by pre-miR-146b-3p was almost abolished in the MAP3K10-MUT mutant, suggesting that the conserved region was fully responsible for miR-146b-3p function. These data suggested that miR-146b-3p directly recognizes the 3′-UTR of MAP3K10 mRNA and inhibited MAP3K10 translation **(Fig. 5I)**. Thus, downregulated miR-146b-3p in pancreatic cancer inhibits the suppression of MAP3K10, which may in turn accelerate growth and tumorigenesis.

### Re-expression of miR-146b-3p regulates the expression of DYRK2 and GLI2

**Figure 6:**
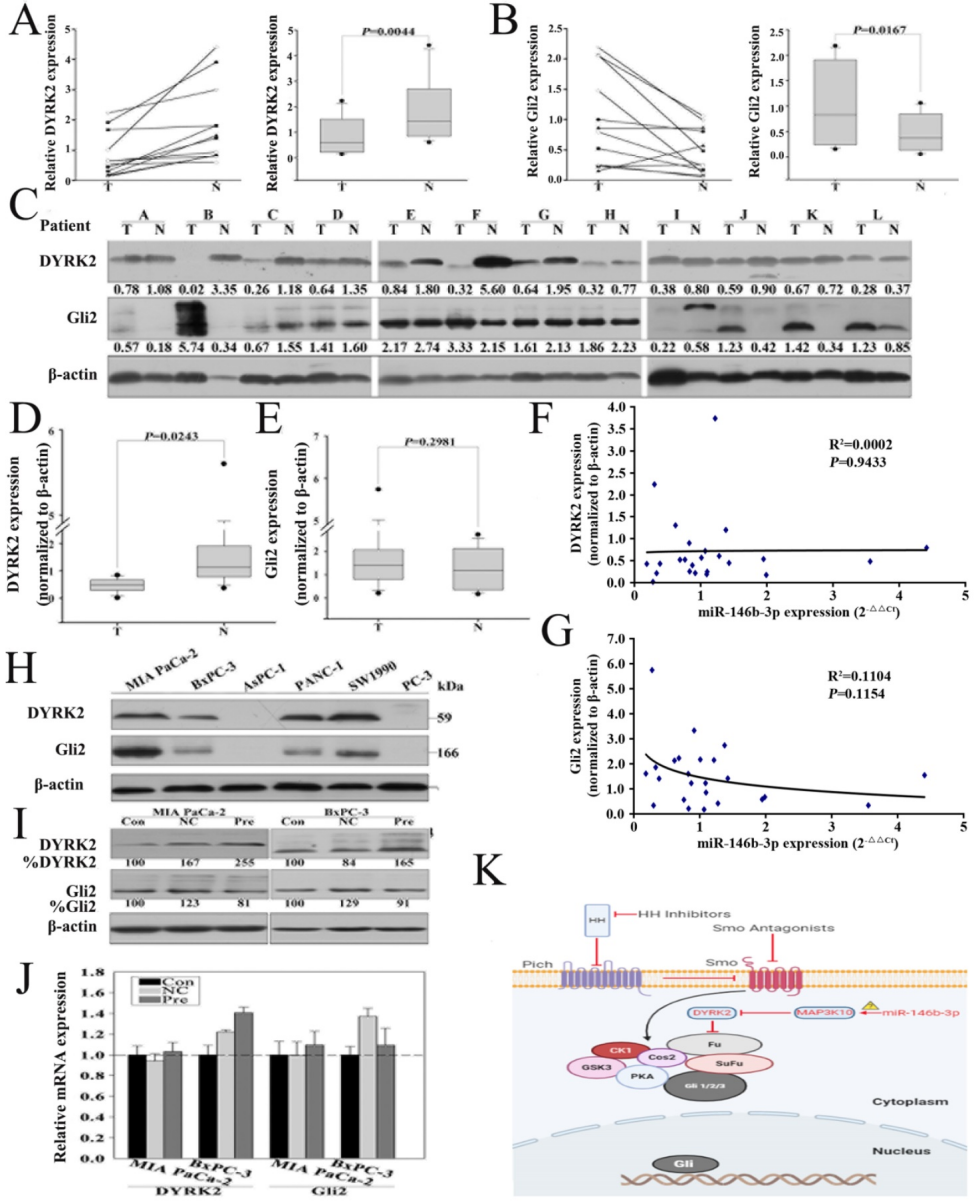
** Re-expression of miR-146b-3p regulates the expression of DYRK2 and GIL2. A) and B)**, Analysis of the gene expression levels of DYRK2 (A) and GIL2 (B) by real time PCR in 12 matched normal pancreatic tissue and pancreatic cancer tissues and (right) the data were analyzed using the Mann-Whitney U test. T, pancreatic cancer tumor tissue; N, pair-matched adjacent non-tumor tissue. **C)** Analysis of the protein expression levels of DYRK2 and GIL2 by western blotting in 12 matched normal pancreatic tissue and pancreatic cancer tissues and (**D and E**) the data were analyzed using the Mann-Whitney U test. T, pancreatic cancer tumor tissue; N, pair-matched adjacent non-tumor tissue. **F and G)** Correlation of the protein expression of DYRK2 and GIL2 with miR-146b-3p expression in 12 pairs of pancreatic cancer tissues and matched normal pancreatic tissues. Coefficient of determination from logarithmic regression model (R2) and P values are given. **H)** Analysis of the protein expression levels of DYRK2 and GIL2 in six pancreatic cancer cell lines by western blotting. **I)** Analysis of Gene expression of DYRK2 and GIL2 in Mia PaCa-2 and BxPC-3 cells after transfection with miR-146b-3p mimic or mimic NC or no transfection by real time PCR. **J)** Analysis of protein expression of DYRK2 and GIL2 in Mia PaCa-2 and BxPC-3 cells after transfection with miR-146b-3p mimic or mimic NC or no transfection by western blot. **K)** Working model for miR-146b-3p-MAP3K10-Gli2 axis.

MAP3K10 was demonstrated to regulate GLI2, the key Hh pathway-regulated transcription factor, indirectly through modulating the activity of DYRK2** (Fig. 6K)**. To confirm that re-expression of miR-146b-3p can regulate the expression of DYRK2 and GIL2, we first detected DYRK2 and GIL2 in the 12 matched normal and cancer tissues by RT-PCR and western blot. We found that DYRK2 gene and protein was markedly low abundant in cancer tissues **(Fig. 6A, C and D)**, but GIL2 was just distinct stronger in gene lever, not in protein lever **(Fig. 6B, C and E)**. Then, we did not find a reversed nonlinear correlation between the level of the DYRK2 and GIL2 protein and miR-146b-3p expression **(Fig. 6F, G)**. We also detected DYRK2 and GIL2 protein in five pancreatic cancer cell lines **(Fig. 6H)**. The expression of DYRK2 and GIL2 after transfection with the pre-miR-146b-3p was detected by RT-PCR and western blot. We found DYRK2 was upregulated and GLI2 was downregulated in pre-miR-146b-3p-treated MIA-PaCa-2 cells at protein level, but not at mRNA level **(Fig. 6I, J)**.

## Discussion

For the first time, we have profiled the genome-wide and the miRNA expression patterns in purified subpopulations (CD24+CD44+ESA+) that possess stem/progenitor cell properties. The side populations isolated from two pancreatic tumor xenograft are highly tumorigenic, clonogenic and significantly drug-resistant. Micro-array analysis revealed 479 genes and 15 miRNAs to be differentially expressed in P-CSCs and non-P-CSCs. We then demonstrated that miR-146b-3p regulated cell growth and tumorigenesis in P-CSCs, but nor or slightly in non- P-CSCs, and may regulate the Hedgehog signaling pathway by directly targets MAP3K10. Taken together, our findings demonstrate that several key signaling pathways, such as the p53- and TGF-β-signaling pathways, and some distinct miRNA expression patterns may coordinately regulate the stem cell properties of pancreatic cancer cells.

Although there are abundant data examining gene expression profiles of normal and malignant bulk cell populations in pancreatic cancer, little has been done to apply the stem cell model to analyze gene expression in pancreatic cancer samples 21, 22. To clarify the molecular mechanisms leading to stem cell-like properties of P-CSCs, we used genomic and miRNA profiling of P-CSCs to identify some aberrantly expressed genes and pathways. By GO analysis, gene-net analysis and pathway-net analysis, we demonstrated that *EGFR*, *SMAD3*, *RHOA*, *TGFBR2*, *CD44*, *CTNNB1*, *MAPK1* and *PTK2* may be the crucial genes, and that the p53- and TGF-β-signaling pathways may be critical to P-CSCs. TGF-β is a cytokine with a dual role in cancer, acting as a tumor suppressor in normal epithelial cells and early-stage tumors, but becoming an oncogenic factor in advanced tumors, inducing proliferation, angiogenesis, invasion, suppression of the immune response, and metastasis 23, 24. Although the TGF-β pathway has been shown to play a broad and multifunctional role in pancreatic carcinogenesis, the function of it in pancreatic cancer-initiating cells is still unknown 25, 26. Reportedly, autocrine TGF-β signaling plays an essential role in retention of stemness of glioma-initiating cells through Sry-related HMG-box factors; TGF-β has also been shown to increase glioma-initiating cell self-renewal through induction of LIF in human glioblastoma 27, 28. Moreover, it was reported that TGF-β can suppress tumorigenesis through effects on putative CSCs or early progenitor cells and their progeny in a breast cancer xenograft model 29. These data suggest crucial roles for the TGF-β pathway in regulating tumor-initiating cells 30. We found that *TGFBR2* and *SMAD3*, the key genes of TGF-β pathway, are up-regulated in P-CSCs compared with their progeny cells. These data suggest that strategies affecting the TGF-β signaling pathway in P-CSCs offer novel forms of differentiation therapy for pancreatic tumors.

Recent evidence indicates the critical role of miRNAs in regulation of various biological and pathologic processes, including stem cell properties. More importantly, it has been recently suggested that aberrant up- and down-regulation of specific miRNAs and their targets in various types of cancer be associated with development and progression of cancer 31, 32. Reportedly, significant differences were found between tumors and chronic pancreatitis, normal pancreas, pancreatic cellular lines, and adjacent normal samples 33. These findings suggest that miRNA expression patterns constitute a signature of the disease that could offer new clues about pancreatic cancer occurrence, while providing molecular markers that would improve diagnosis and treatment tools 31. More recently, reports of our research and other studies have highlighted the importance of miRNAs to maintain the stemness of tumor-initiating cells 34. Song found that *let-7* regulates the key features of breast-CSC self-renewal *in vitro*, multipotent differentiation, and the ability to form tumors that can be serially transplanted and metastasize in NOD/SCID mice 3. Xu et al. showed that *miR-34* may be involved in P-CSC self-renewal, via direct modulation of downstream targets Bcl-2 and Notch, implying that *miR-34* plays an important role in P-CSC self-renewal and/or cell fate determination 7. Here, we first identified 15 miRNAs that were aberrantly expressed in P-CSCs, including miRNAs previously reported as differentially expressed in pancreatic cancer and/or other human cancers (*miR-146b*, *miR-424*, and *miR-20a*), and others not previously associated with cancer (*miR-590-3p* and *miR-575*).

MiR-146b gene is located in chromosome 10 (104186259-104186331). Recently, it has been shown to be associated with some cancers and plays a critical role in tumor progression. Although there has been evidence showing that miR-146b mediates cancer metastasis, its function in regulating cancer stem cells properties remains unexplored 35, 36. We found miR-146b-3p was remarkably downregulated in P-CSCs and played a more significant regulating function in Mia-paca-2 (CSC^high^) than in BxPC3 (CSC^low^). To investigate whether miR-146b-3p affected P-CSCs through the Hh pathway, a crucial role for activated Shh signaling in pancreatic cancer (stem) cells, we choose MAP3K10 as the potential targeted gene. It has been reported MAP3K10 could modulate the activity of DYRK2, which directly phosphorylated and induced the proteasome-dependent degradation of the key Hh pathway-regulated transcription factor, GLI2 37. A study revealed that DYRK2 directly phosphorylated and induced degradation of GLI2. It is reported that MAP3K10 plays an important role in the tumorigenesis and survival of pancreatic cancers by upregulation of Gli-1 and Gli-2, accompanied by decreased expression of DYRK2 38, 39. Jimeno et al and Mueller et al confirmed that the combined blockade of sonic hedgehog with standard chemotherapy is capable of eliminating pancreatic CSCs 18. Recently, these reports also demonstrated that combined blockade yet could not eradicate pancreatic CSCs. Our data showed that miR-146b-3p restoration directly down-regulated the expression of MAP3K10, which may regulate the activation of Hedgehog pathway by DYRK2 and GLI2. Almost all blockade agents of sonic hedgehog inhibited the protein upstream of MAP3K10, we speculated that the abnormal activation of Hedgehog pathway by miR-146b-3p might the reason that pancreatic CSCs escaped the combined blockade. By modulating CSCs, the restoration of miR-146b-3p may provide a novel therapeutic approach for pancreatic cancer.

In summary, this study showed genomic and miRNA profiling of P-CSCs, and identified several potential key genes and pathways. Further investigations of these aberrant genes and pathways may elucidate molecular mechanisms of uncontrolled self-renewal, aberrant differentiation, and chemoresistance of P-CSCs. New therapies targeted against CSCs may lead to improved cancer treatments, preventing disease relapse, and potentially inducing complete disease remissions.

## Supplementary Material

Supplementary figures and tables.

## Figures and Tables

**Table 1:** Top 50 differentially up- and down-regulated genome-wide genes and all different microRNAs between P-CSCs and Non-P-CSCs

Gene Title	Gene Symbol	Log Ratio Case 1	Log Ratio Case 2	Gene Title	Gene Symbol	Log Ratio Case 1	Log Ratio Case 2
CD55 molecule	*CD55*	6.2	0.7	cadherin 11, type 2, OB-cadherin	*CDH11*	-8.9	-0.6
chromosome 1 open reading frame 119	*C1orf119*	5.8	0.7	glycoprotein M6B	*GPM6B*	-8.1	-1.7
Dystonin	*DST*	5.4	1	runt-related transcription factor	*RUNX1T1*	-7.9	-2.1
matrix metallopeptidase 1	*MMP1*	5.3	0.6	nuclear factor I/B	*NFIB*	-7.8	-3.5
chitobiase, di-N-acetyl-	*CTBS*	5.3	0.9	purinergic receptor P2Y, G-protein coupled, 5	*P2RY5*	-7.5	-3.1
Mesothelin	*MSLN*	5.1	0.6	cadherin 10, type 2	*CDH10*	-7.4	-0.8
chromosome 17 open reading frame 91	*C17orf91*	5	0.8	GLI-Kruppel family member GLI3	*GLI3*	-7.4	-1
suppressor of cytokine signaling 4	*SOCS4*	5	0.8	Zic family member 4	*ZIC4*	-7.4	-1.4
junction plakoglobin	*JUP*	4.9	0.6	chromosome 7 open reading frame 4	*C7orf4*	-7.3	-0.8
ATP-binding cassette, sub-family C	*ABCC3*	4.9	0.8	synaptonemal complex protein 1	*SYCP1*	-7	-6
occludin /// occludin pseudogene	*LOC647859*	4.9	0.6	B-cell CLL/lymphoma 11B	*BCL11B*	-6.5	-1.2
selenoprotein P, plasma, 1	*SEPP1*	4.8	0.7	Boc homolog (mouse)	*BOC*	-6.4	-1.1
tetratricopeptide repeat domain 12	*TTC12*	4.8	1.8	ARP3 actin-related protein 3 homolog B pseudogene	*LOC644773*	-6.4	-4.5
CDC14 cell division cycle 14 homolog B	*CDC14B*	4.8	0.8	LIM and senescent cell antigen-like domains 1	*LIMS1*	-6.3	-1.3
hypothetical protein FLJ31033	*FLJ31033*	4.8	2.5	zinc finger protein 423	*ZNF423*	-6.3	-0.7
jumonji, AT rich interactive domain 1B	*JARID1B*	4.7	0.7	hypothetical gene supported by BC037858	*LOC440117*	-6.2	-1.2
DnaJ (Hsp40) homolog, subfamily A, member 4	*DNAJA4*	4.6	0.9	eukaryotic translation initiation factor 5A	*EIF5A*	-6	-1.5
myosin VA (heavy chain 12, myoxin)	*MYO5A*	4.6	0.6	keratin associated protein 19-1	*KRTAP19-1*	-6	-4.4
protein inhibitor of activated STAT, 3	*PIAS3*	4.5	0.7	dermatopontin	*DPT*	-5.9	-1.7
interferon-induced protein with tetratricopeptide repeats 1	*IFIT1*	4.5	0.8	hypothetical protein LOC285419	*LOC285419*	-5.9	-2.7
pyruvate kinase, muscle	*PKM2*	4.4	1.9	SEC14-like 4	*SEC14L4*	-5.9	-2.8
PQ loop repeat containing 3	*PQLC3*	4.4	2	ectonucleotide pyrophosphatase/phosphodiesterase 2	*ENPP2*	-5.7	-2.6
guanylate binding protein 3	*GBP3*	4.3	0.7	ropporin, rhophilin associated protein 1	*ROPN1*	-5.7	-1.1
cytochrome P450, family 4, subfamily X, polypeptide 1	*CYP4X1*	4.3	0.8	chromosome 5 open reading frame 13	*C5orf13*	-5.6	-0.6
O-linked N-acetylglucosamine (GlcNAc) transferase	*OGT*	4.2	0.7	ATPase, H+ transporting, lysosomal 38kDa, V0 subunit d2	*ATP6V0D2*	-5.5	-1.1
hsa-miR-590-3p		2.49	2.35	hsa-miR-146b-3p		-2.40	-3.00
hsa-miR-20a*		1.28	1.35	hsa-miR-575		-1.57	-1.70
hsa-miR-142-3p		0.92	1.66	hsa-miR-647		-1.21	-2.30
hsa-miR-424		0.75	1.20	hsa-miR-638		-1.05	-0.69
hsa-miR-29a*		0.66	1.20	hsa-miR-494		-0.97	-0.79
hsa-miR-135b		0.65	1.11	hsa-miR-939		-0.83	-1.11
hsa-miR-141*		0.60	1.50	hsa-miR-1226*		-0.76	-1.07
				hsa-miR-423-3p		-0.70	-1.75

## References

[1] Siegel R, Ma J, Zou Z, Jemal A (2014). Cancer statistics, 2014. CA: a cancer journal for clinicians.

[2] Li C, Heidt DG, Dalerba P, Burant CF, Zhang L, Adsay V (2007). Identification of pancreatic cancer stem cells. Cancer research.

[3] Yu F, Yao H, Zhu P, Zhang X, Pan Q, Gong C (2007). let-7 regulates self-renewal and tumorigenicity of breast cancer cells. Cell.

[4] Hermann PC, Huber SL, Herrler T, Aicher A, Ellwart JW, Guba M (2007). Distinct populations of cancer stem cells determine tumor growth and metastatic activity in human pancreatic cancer. Cell stem cell.

[5] Hatfield SD, Shcherbata HR, Fischer KA, Nakahara K, Carthew RW, Ruohola-Baker H (2005). Stem cell division is regulated by the microRNA pathway. Nature.

[6] Bartel DP (2004). MicroRNAs: genomics, biogenesis, mechanism, and function. Cell.

[7] Ji Q, Hao X, Zhang M, Tang W, Yang M, Li L (2009). MicroRNA miR-34 inhibits human pancreatic cancer tumor-initiating cells. PloS one.

[8] Bao B, Ali S, Ahmad A, Li Y, Banerjee S, Kong D (2014). Differentially Expressed miRNAs in Cancer-Stem-Like Cells: Markers for Tumor Cell Aggressiveness of Pancreatic Cancer. Stem cells and development.

[9] DeSano JT, Xu L (2009). MicroRNA regulation of cancer stem cells and therapeutic implications. The AAPS journal.

[10] Bartel DP (2009). MicroRNAs: target recognition and regulatory functions. Cell.

[11] Tang SN, Fu J, Nall D, Rodova M, Shankar S, Srivastava RK (2012). Inhibition of sonic hedgehog pathway and pluripotency maintaining factors regulate human pancreatic cancer stem cell characteristics. International journal of cancer Journal international du cancer.

[12] Singh BN, Kumar D, Shankar S, Srivastava RK (2012). Rottlerin induces autophagy which leads to apoptotic cell death through inhibition of PI3K/Akt/mTOR pathway in human pancreatic cancer stem cells. Biochemical pharmacology.

[13] Liu L, Salnikov AV, Bauer N, Aleksandrowicz E, Labsch S, Nwaeburu C (2014). Triptolide reverses hypoxia-induced epithelial-mesenchymal transition and stem-like features in pancreatic cancer by NF-kappaB downregulation. International journal of cancer Journal international du cancer.

[14] Rausch V, Liu L, Kallifatidis G, Baumann B, Mattern J, Gladkich J (2010). Synergistic activity of sorafenib and sulforaphane abolishes pancreatic cancer stem cell characteristics. Cancer research.

[15] Rodova M, Fu J, Watkins DN, Srivastava RK, Shankar S (2012). Sonic hedgehog signaling inhibition provides opportunities for targeted therapy by sulforaphane in regulating pancreatic cancer stem cell self-renewal. PloS one.

[16] Zhang JD, Wiemann S (2009). KEGGgraph: a graph approach to KEGG PATHWAY in R and bioconductor. Bioinformatics.

[17] Guo CJ, Pan Q, Li DG, Sun H, Liu BW (2009). miR-15b and miR-16 are implicated in activation of the rat hepatic stellate cell: An essential role for apoptosis. Journal of hepatology.

[18] Jimeno A, Feldmann G, Suarez-Gauthier A, Rasheed Z, Solomon A, Zou GM (2009). A direct pancreatic cancer xenograft model as a platform for cancer stem cell therapeutic development. Molecular cancer therapeutics.

[19] Draghici S, Khatri P, Tarca AL, Amin K, Done A, Voichita C (2007). A systems biology approach for pathway level analysis. Genome research.

[20] Merchant AA, Matsui W (2010). Targeting Hedgehog-a cancer stem cell pathway. Clin Cancer Res.

[21] Wang WY, Hsu CC, Wang TY, Li CR, Hou YC, Chu JM (2013). A gene expression signature of epithelial tubulogenesis and a role for ASPM in pancreatic tumor progression. Gastroenterology.

[22] Jones S, Zhang X, Parsons DW, Lin JC, Leary RJ, Angenendt P (2008). Core signaling pathways in human pancreatic cancers revealed by global genomic analyses. Science.

[23] Qin J, Wu SP, Creighton CJ, Dai F, Xie X, Cheng CM (2013). COUP-TFII inhibits TGF-beta-induced growth barrier to promote prostate tumorigenesis. Nature.

[24] Gore AJ, Deitz SL, Palam LR, Craven KE, Korc M (2014). Pancreatic cancer-associated retinoblastoma 1 dysfunction enables TGF-beta to promote proliferation. The Journal of clinical investigation.

[25] Ellermeier J, Wei J, Duewell P, Hoves S, Stieg MR, Adunka T (2013). Therapeutic efficacy of bifunctional siRNA combining TGF-beta1 silencing with RIG-I activation in pancreatic cancer. Cancer research.

[26] Kabashima A, Higuchi H, Takaishi H, Matsuzaki Y, Suzuki S, Izumiya M (2009). Side population of pancreatic cancer cells predominates in TGF-beta-mediated epithelial to mesenchymal transition and invasion. International journal of cancer Journal international du cancer.

[27] Penuelas S, Anido J, Prieto-Sanchez RM, Folch G, Barba I, Cuartas I (2009). TGF-beta increases glioma-initiating cell self-renewal through the induction of LIF in human glioblastoma. Cancer cell.

[28] Ikushima H, Todo T, Ino Y, Takahashi M, Miyazawa K, Miyazono K (2009). Autocrine TGF-beta signaling maintains tumorigenicity of glioma-initiating cells through Sry-related HMG-box factors. Cell stem cell.

[29] Bierie B, Chung CH, Parker JS, Stover DG, Cheng N, Chytil A (2009). Abrogation of TGF-beta signaling enhances chemokine production and correlates with prognosis in human breast cancer. The Journal of clinical investigation.

[30] Qin H, Qu C, Yamaza T, Yang R, Lin X, Duan XY (2013). Ossifying fibroma tumor stem cells are maintained by epigenetic regulation of a TSP1/TGF-beta/SMAD3 autocrine loop. Cell stem cell.

[31] Li Z, Rana TM (2014). Therapeutic targeting of microRNAs: current status and future challenges. Nature reviews Drug discovery.

[32] Croce CM, Calin GA (2005). miRNAs, cancer, and stem cell division. Cell.

[33] Bloomston M, Frankel WL, Petrocca F, Volinia S, Alder H, Hagan JP (2007). MicroRNA expression patterns to differentiate pancreatic adenocarcinoma from normal pancreas and chronic pancreatitis. JAMA: the journal of the American Medical Association.

[34] Shimono Y, Zabala M, Cho RW, Lobo N, Dalerba P, Qian D (2009). Downregulation of miRNA-200c links breast cancer stem cells with normal stem cells. Cell.

[35] Geraldo MV, Yamashita AS, Kimura ET (2012). MicroRNA miR-146b-5p regulates signal transduction of TGF-beta by repressing SMAD4 in thyroid cancer. Oncogene.

[36] Liao Y, Zhang M, Lonnerdal B (2013). Growth factor TGF-beta induces intestinal epithelial cell (IEC-6) differentiation: miR-146b as a regulatory component in the negative feedback loop. Genes & nutrition.

[37] Varjosalo M, Bjorklund M, Cheng F, Syvanen H, Kivioja T, Kilpinen S (2008). Application of active and kinase-deficient kinome collection for identification of kinases regulating hedgehog signaling. Cell.

[38] An Y, Cai B, Chen J, Lv N, Yao J, Xue X (2013). MAP3K10 promotes the proliferation and decreases the sensitivity of pancreatic cancer cells to gemcitabine by upregulating Gli-1 and Gli-2. Cancer letters.

[39] Gallo KA, Johnson GL (2002). Mixed-lineage kinase control of JNK and p38 MAPK pathways. Nature reviews Molecular cell biology.

