# Supplementary material for: MiR-146b-3p regulates proliferation of pancreatic cancer cells with stem cell-like properties by targeting MAP3K10
Source: J Cancer. 2021 May 3;12(12):3726–40. doi: 10.7150/jca.48418 (PMC8120187; doi:10.7150/jca.48418)
Supplement: Supplementary file 1 — Supplementary figures and tables. [file jcav12p3726s1.pdf]

Supplementary Figures and Tables

Supplementary Figures:

Figure S1. Analysis of the dysregulated genes in TGF-β (A) and p53 (B) signaling pathway by the KEGG data base. The dysregulated gene is in red.

Figure S2. Detection of the expressions of 16 microRNAs, including 15 microRNA identified by microarray and miR-146b-5p (G), in 12 pairs of pancreatic cancer tissues and matched normal pancreatic tissues by RT-PCR.

Figure S3. Detection of the expressions of 16 microRNAs, including 15 microRNA identified by microarray and miR-146b-5p, in five pancreatic cancer cell lines and normal pancreatic tissues by RT-PCR.

Figure S4. Over-expression of miR-146b-3p affected cell growth, cell cycle, apoptosis. A) Growth of MIA PaCa-2 and PANC-1 cells was shown after transfection with miR-146b-3p mimic or mimic NC or no transfection. The growth index was assessed at 1, 2, and 3 d by the CSFE assay.B) Over-expression of miR-146b-3p resulted in G1 arrest and S-phase increasing in Mia PaCa-2 and not in BxPC-3 cells as measured by PI staining.C) Apoptosis of MIA PaCa-2 and PANC-1 cells were detected by Annexin V/PI after treatment with miR-146b-3p mimic or mimic NC or no transfection in 72 h. All data are shown as mean ± SD. *, $P < 0.05$; **, $P < 0.01$.

Figure S1

A

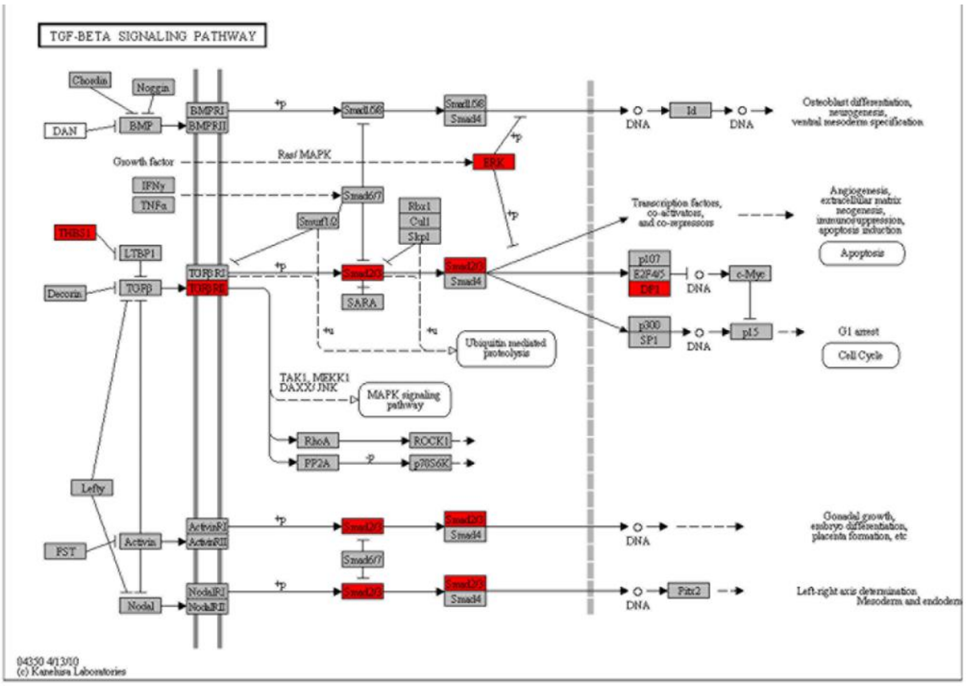
TGF-BETA SIGNALING PATHWAY
Chordin
Noggin
DAN
BMP
BMPRI
BMPRII
Smad1/5/8
Smad4
Id
DNA
Osteoblast differentiation, neurogenesis, ventral mesoderm specification
Growth factor
Ras/ MAPK
ERK
IFNγ
TNFα
Smad6/7
Smurf1/2
Rbx1
Cul1
Skp1
THBS1
LTBP1
Decorin
TGFβ
TGFβRI
TGFβRII
Smad2/3
SARA
Transcription factors, co-activators, and co-repressors
Angiogenesis, extracellular matrix neogenesis, immunosuppression, apoptosis induction
Apoptosis
p107
E2F4/5
DP1
c-Myc
p300
SP1
p15
G1 arrest
Cell Cycle
Ubiquitin mediated proteolysis
TAK1, MEKK1
DAXX/JNK
MAPK signaling pathway
RhoA
ROCK1
PP2A
p70S6K
Lefty
FST
Activin
ActivinRI
ActivinRII
Gonadal growth, embryo differentiation, placenta formation, etc
Nodal
NodalRI
NodalRII
Pitx2
Left-right axis determination
Mesoderm and endoderm
04350 4/13/10
(c) Kanehisa Laboratories

B

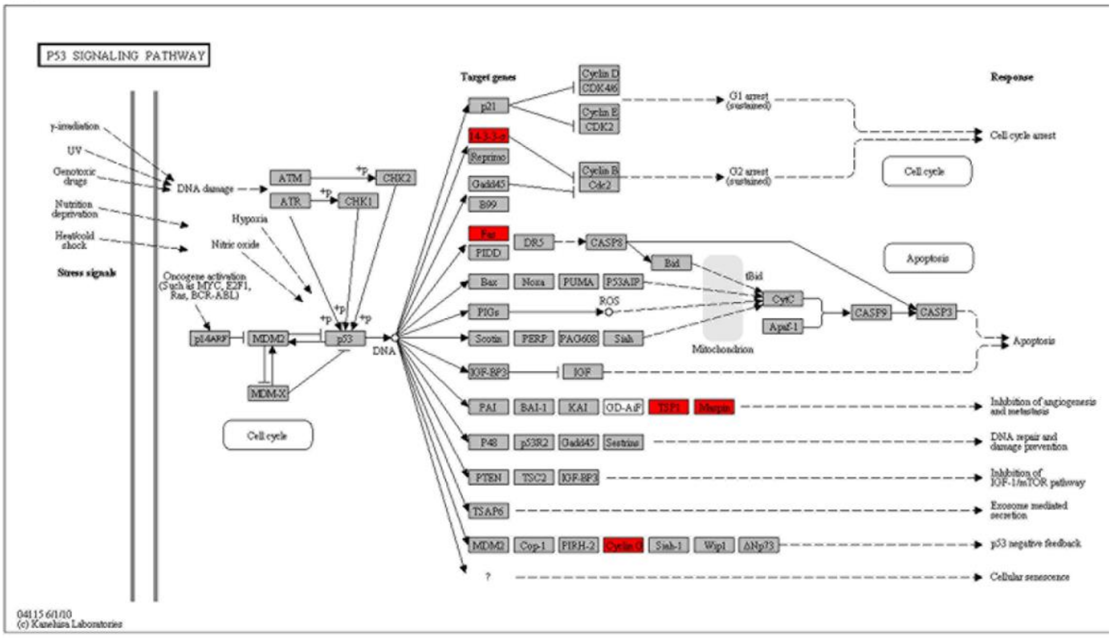
P53 SIGNALING PATHWAY
Target genes
Response
γ-irradiation
UV
Genotoxic drugs
Nutrition deprivation
Heat/cold shock
Stress signals
DNA damage
ATM
ATR
CHK2
CHK1
Hypoxia
Nitric oxide
Oncogene activation (Such as MYC, E2F1, Ras, BCR-ABL)
p14ARF
MDM2
MDM-X
p53
DNA
Cell cycle
p21
14-3-3-σ
Reprimo
Gadd45
B99
Cyclin D
CDK4/6
Cyclin E
CDK2
Cyclin B
Cdc2
G1 arrest (sustained)
G2 arrest (sustained)
Cell cycle arrest
Cell cycle
Fas
PIDD
DR5
CASP8
Bid
tBid
Bax
Noxa
PUMA
P53AIP
PIGs
ROS
Scotin
PERP
PAG608
Siah
CytC
Apaf-1
CASP9
CASP3
Mitochondrion
Apoptosis
IGF-BP3
IGF
PAI
BAI-1
KAI
GD-AiF
TSP1
Maspin
Inhibition of angiogenesis and metastasis
P48
p53R2
Gadd45
Sestrins
DNA repair and damage prevention
PTEN
TSC2
IGF-BP3
Inhibition of IGF-1/mTOR pathway
TSAP6
Exosome mediated secretion
MDM2
Cop-1
PIRH-2
Cyclin G
Siah-1
Wip1
ΔNp73
p53 negative feedback
Cellular senescence
04115 6/1/10
(c) Kanehisa Laboratories

Figure S2

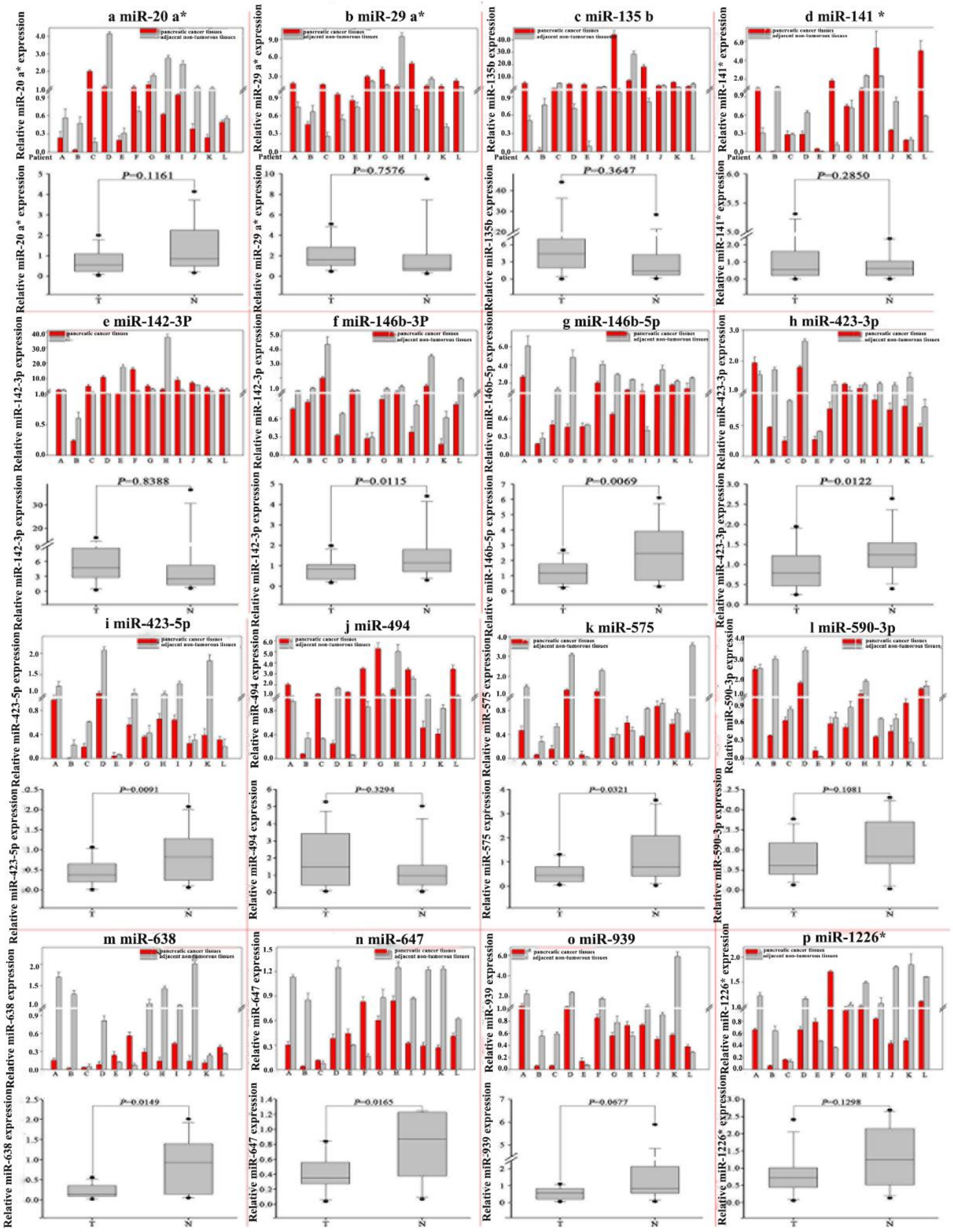
a miR-20 a*
b miR-29 a*
c miR-135 b
d miR-141 *
P=0.1161
P=0.7576
P=0.3647
P=0.2850
e miR-142-3P
f miR-146b-3P
g miR-146b-5p
h miR-423-3p
P=0.8388
P=0.0115
P=0.0069
P=0.0122
i miR-423-5p
j miR-494
k miR-575
l miR-590-3p
P=0.0091
P=0.3294
P=0.0321
P=0.1081
m miR-638
n miR-647
o miR-939
p miR-1226*
P=0.0149
P=0.0165
P=0.0677
P=0.1298
pancreatic cancer tissues
adjacent non-tumorous tissues

Figure S3

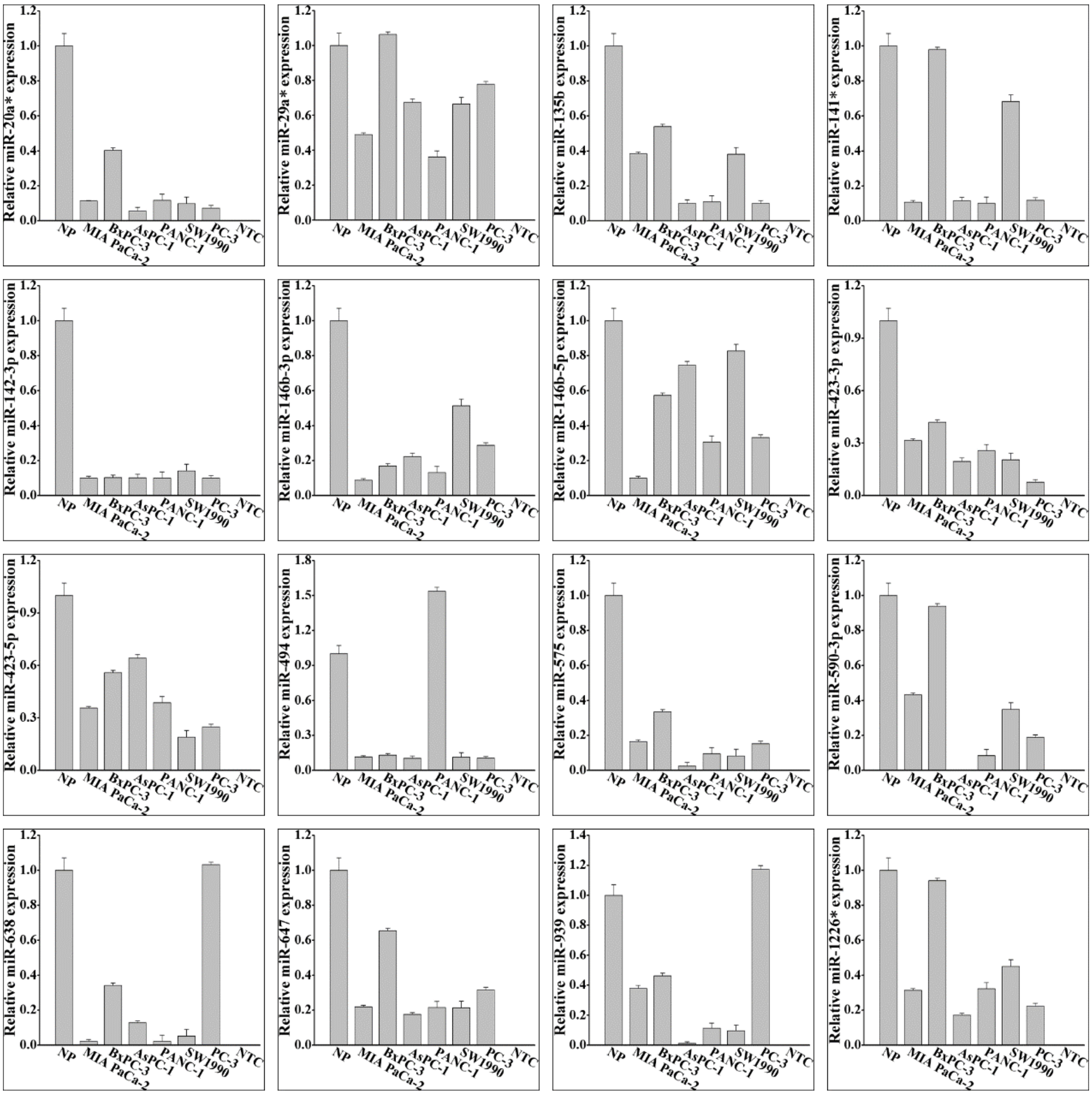

Relative miR-20a* expression
Relative miR-29a* expression
Relative miR-135b expression
Relative miR-141* expression
Relative miR-142-3p expression
Relative miR-146b-3p expression
Relative miR-146b-5p expression
Relative miR-423-3p expression
Relative miR-423-5p expression
Relative miR-494 expression
Relative miR-575 expression
Relative miR-590-3p expression
Relative miR-638 expression
Relative miR-647 expression
Relative miR-939 expression
Relative miR-1226* expression
NP
MIA PaCa-2
BxPC-3
AsPC-1
PANC-1
SW1990
PC-3
NTC

Figure S4

A

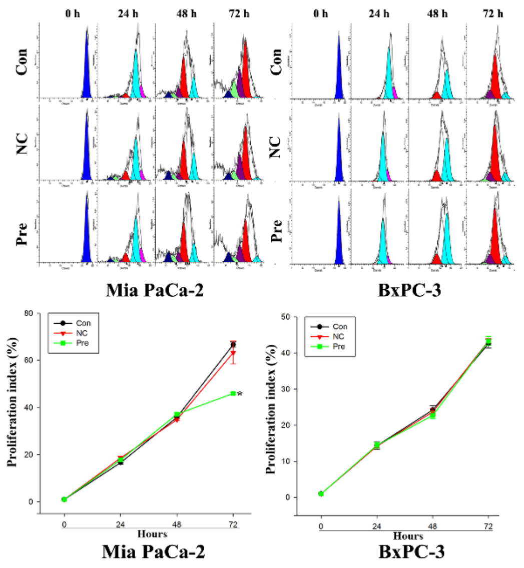

B

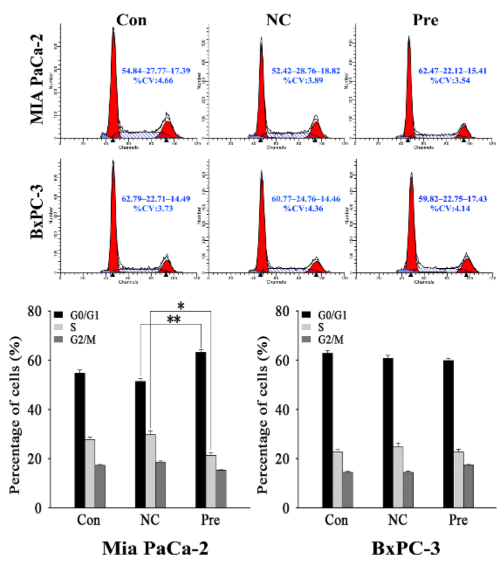

C

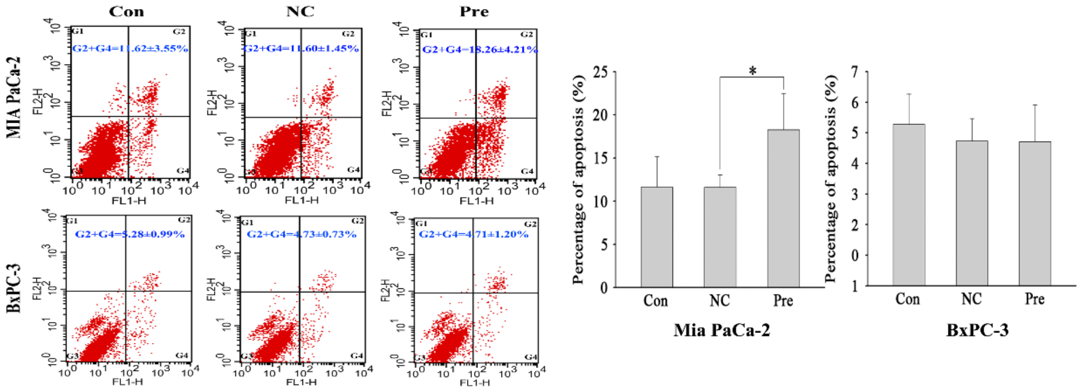

Supplemental table 1. antibody used for western blot and Flow cytometry

**Name**	**company**	**clone No.**
Rabbit polyclonal to human DYRK2	Abcam (Hong Kong)Ltd	ab37912
Rabbit polyclonal to human MLK2(MAP3K10)	Abcam (Hong Kong)Ltd	ab70859
Sheep monoclonal to human GLI-2	R&D SYSTEMS, Inc	AF3526
Mouse monoclonal to human cyclin D1	SANTA CRUZ BIOTECHNOLOGY,INY	sc-8396
Rabbit polyclonal to human Cyclin E1(CCNE1)	ProteinTech Group, Inc	11554-1-AP
Mouse monoclonal to human p27	SANTA CRUZ BIOTECHNOLOGY,INY	sc-1641
Mouse monoclonal to human Bcl-2	SANTA CRUZ BIOTECHNOLOGY,INY	sc-492
Rabbit monoclonal to human BAX	SANTA CRUZ BIOTECHNOLOGY,INY	sc-526
Mouse monoclonal to human caspase-3	SANTA CRUZ BIOTECHNOLOGY,INY	sc-7148
Rabbit polyclonal to human Bcl-xl	ProteinTech Group, Inc	10783-1-AP
Rabbit polyclonal to human caspase-9(CASP9)	ProteinTech Group, Inc	10380-1-AP
Rabbit polyclonal to human caspase-8(CASP8)	ProteinTech Group, Inc	11554-1-AP
Mouse monoclonal to human β-Actin	SANTA CRUZ BIOTECHNOLOGY,INY	sc-130301
Mouse monoclonal to human ESA-FITC	Biomeda	B29.1
Goat monoclonal to mouse H2kd-PE	BD Biosciences PharMingen	553566
Mouse monoclonal to human CD24-PE	BD Biosciences PharMingen	555428
Mouse monoclonal to human CD24-FITC	BD Biosciences PharMingen	555427
Mouse monoclonal to human CD44-APC	BD Biosciences PharMingen	559942
Mouse monoclonal to human EpCAM-PerCP-Cy5.5	BD Biosciences	347199

Supplemental table 2. Primers and conditions used for RT-PCR and qPCR.

**Gene**	**RT-PCR**	**qPCR**
SMAD3		5′-GTCGGTCAACCAGGGCTTT-3′
		5'-CCGCTCCCCAGCCTTT-3
SHH	5'-CGCACGGGGACAGCTCGGAAGT-3'	
	5'-CTGCGCGGCCCTCGTAGTGC-3'	
PTCH	5'-GCCGTGCCCGTGGTCAT-3'	
	5'-CCCATTGAGAACGCCGAGGAT-3'	
SMO	5'-GAAGTGCCCTTGGTTCGGACA-3'	
	5'-CCGCCAGTCAGCCACGAAT-3'	
MAP3K10		5'-ACGGAATTCATGGAGGAGGAGGAGGGGGCGGTGG-3'
		5'-ACGCTCGAGTTAGTGGGAGCCGTGGGCCCCGCAC-3'
DYRK2		5'-GCCATGTTAACCAGGAAACC-3'
		5'-GAAGCCTGAAGCTGACGAAC-3'
GLI1	5'-CAGGGAGTGCAGCCAATACAG-3'	
	5'-GAGCGGCGGCTGACAGTATA-3'	
GLI2	5'-AGAAGCAGCGCAATGACGTG-3'	5'-TGGCCGCTTCAGATGACAGATGTTG-3'
	5'-GTCATCCAGTGCCGTCAGGT-3'	5'-CGTTAGCCGAATGTCAGCCGTGAAG-3'
GAPDH		5′-GGGTGTGAACCATGAGAAGT-3′
		5′ -GGCATGGAC TGTGGTCATGA-3′
MAP3K10	5’-GATCGCCGTGTAAT***TCTAGA***GGCCTGCCCACCACC -3’ (XbaI sites are in bold and italic)	
3’UTR	5’- CCGGCCGCCCCGAC***TCTAGA***AGGGGGCAGGGGTTACC- 3’	
MAP3K10	5’- GATCGCCGTGTAAT***TCTAGA***GGCCTGCCCACCACCGCCCGCCTG-3’ (XbaI sites are in bold and italic)	
3'UTR-mut	5’-CCGGCCGCCCCGAC***TCTAGA***AGGGGGCAGGGGTTACCTTTAATGCTCCATTTTGTGCC -3’	

Supplemental table 3. Go analysis of the dysregulated genes identified by microarray

go_id	go_name	pvalue	FDR	enrichment	go_diff gene_count	go_gene_count
**genes up-regulated**						
GO:0007049	cell cycle	0.0001123	0.009321	2.528747	23	769
GO:0006511	ubiquitin-dependent protein catabolic process	0.0001495	0.010055	2.774463	19	579
GO:0042981	regulation of apoptosis	0.0001864	0.010552	4.973419	9	153
GO:0006512	ubiquitin cycle	0.0001866	0.010554	2.927328	17	491
GO:0006468	protein amino acid phosphorylation	0.0003047	0.011684	2.418802	22	769
GO:0006897	endocytosis	0.0003786	0.012084	4.529363	9	168
GO:0030155	regulation of cell adhesion	0.0004015	0.012181	13.5277	4	25
GO:0016477	cell migration	0.0004287	0.012286	7.045676	6	72
GO:0001666	response to hypoxia	0.0004353	0.01231	5.802322	7	102
GO:0015718	monocarboxylic acid transport	0.0005056	0.012997	23.05858	3	11
GO:0006693	prostaglandin metabolic process	0.0008611	0.020164	19.5111	3	13
GO:0060056	mammary gland involution	0.001649	0.032253	42.27406	2	4
GO:0030216	keratinocyte differentiation	0.002075	0.037264	8.899802	4	38
GO:0007050	cell cycle arrest	0.0022418	0.03901	4.416693	7	134
GO:0016056	rhodopsin mediated signaling pathway	0.0027269	0.043522	33.81925	2	5
GO:0006915	apoptosis	0.002863	0.044657	2.179667	19	737
GO:0007067	mitosis	0.0030692	0.046282	3.131412	10	270
GO:0009306	protein secretion	0.0032274	0.047457	12.68222	3	20
GO:0006869	lipid transport	0.0040363	0.052654	4.611715	6	110
GO:0030147	natriuresis	0.0040583	0.052779	28.18271	2	6
GO:0007179	transforming growth factor beta receptor signaling pathway	0.0049849	0.057429	7.045676	4	48
GO:0030195	negative regulation of blood coagulation	0.0056371	0.060113	24.1566	2	7
GO:0008544	epidermis development	0.0057399	0.0605	4.299057	6	118
GO:0001558	regulation of cell growth	0.0075169	0.066009	3.565282	7	166
GO:0000278	mitotic cell cycle	0.0095133	0.070351	18.78847	2	9
GO:0008631	induction of apoptosis by oxidative stress	0.0095133	0.070351	18.78847	2	9
GO:0051899	membrane depolarization	0.0095133	0.070351	18.78847	2	9
GO:0009790	embryonic development	0.0099092	0.071054	5.830905	4	58
GO:0015711	organic anion transport	0.0115592	0.073577	8.182076	3	31
GO:0019835	cytolysis	0.0115592	0.073577	8.182076	3	31
GO:0030146	diuresis	0.0117989	0.073897	16.90962	2	10

GO:0000209	protein polyubiquitination	0.0126443	0.074948	7.926386	3	32
GO:0045766	positive regulation of angiogenesis	0.0126443	0.074948	7.926386	3	32
GO:0006886	intracellular protein transport	0.0139004	0.076317	2.688809	9	283
GO:0000165	MAPKKK cascade	0.0162552	0.078406	7.246981	3	35
GO:0042632	cholesterol homeostasis	0.0162552	0.078406	7.246981	3	35
GO:0008154	actin polymerization or depolymerization	0.0170365	0.078991	14.09135	2	12
GO:0045995	regulation of embryonic development	0.0170365	0.078991	14.09135	2	12
GO:0006631	fatty acid metabolic process	0.01799	0.079647	3.950847	5	107
GO:0042104	positive regulation of activated T cell proliferation	0.0199775	0.080839	13.0074	2	13
GO:0042311	vasodilation	0.0199775	0.080839	13.0074	2	13
GO:0051457	maintenance of protein location in nucleus	0.0199775	0.080839	13.0074	2	13
GO:0007585	respiratory gaseous exchange	0.0204131	0.081074	6.674851	3	38
GO:0030308	negative regulation of cell growth	0.0208795	0.081315	3.808474	5	111
GO:0006916	anti-apoptosis	0.0210021	0.081377	2.694761	8	251
GO:0005975	carbohydrate metabolic process	0.0225325	0.082101	2.348559	10	360
GO:0001569	patterning of blood vessels	0.023126	0.082359	12.0783	2	14
GO:0006479	protein amino acid methylation	0.023126	0.082359	12.0783	2	14
GO:0030301	cholesterol transport	0.023126	0.082359	12.0783	2	14
GO:0002072	optic cup morphogenesis involved in camera-type eye development	0.0236552	0.082579	84.54812	1	1
GO:0002318	myeloid progenitor cell differentiation	0.0236552	0.082579	84.54812	1	1
GO:0002502	peptide antigen assembly with MHC class I protein complex	0.0236552	0.082579	84.54812	1	1
GO:0002651	positive regulation of tolerance induction to self antigen	0.0236552	0.082579	84.54812	1	1
GO:0002663	positive regulation of B cell tolerance induction	0.0236552	0.082579	84.54812	1	1
GO:0002666	positive regulation of T cell tolerance induction	0.0236552	0.082579	84.54812	1	1
GO:0002684	positive regulation of immune system process	0.0236552	0.082579	84.54812	1	1
GO:0006683	galactosylceramide catabolic process	0.0236552	0.082579	84.54812	1	1

GO:0007559	histolysis	0.0236552	0.082579	84.54812	1	1
GO:0009448	gamma-aminobutyric acid metabolic process	0.0236552	0.082579	84.54812	1	1
GO:0019372	lipoxygenase pathway	0.0236552	0.082579	84.54812	1	1
GO:0019377	glycolipid catabolic process	0.0236552	0.082579	84.54812	1	1
GO:0021768	nucleus accumbens development	0.0236552	0.082579	84.54812	1	1
GO:0030003	cellular cation homeostasis	0.0236552	0.082579	84.54812	1	1
GO:0034103	regulation of tissue remodeling	0.0236552	0.082579	84.54812	1	1
GO:0034454	microtubule anchoring at centrosome	0.0236552	0.082579	84.54812	1	1
GO:0042033	chemokine biosynthetic process	0.0236552	0.082579	84.54812	1	1
GO:0042094	interleukin-2 biosynthetic process	0.0236552	0.082579	84.54812	1	1
GO:0042231	interleukin-13 biosynthetic process	0.0236552	0.082579	84.54812	1	1
GO:0042253	granulocyte macrophage colony-stimulating factor biosynthetic process	0.0236552	0.082579	84.54812	1	1
GO:0042313	protein kinase C deactivation	0.0236552	0.082579	84.54812	1	1
GO:0042985	negative regulation of amyloid precursor protein biosynthetic process	0.0236552	0.082579	84.54812	1	1
GO:0043179	rhythmic excitation	0.0236552	0.082579	84.54812	1	1
GO:0045603	positive regulation of endothelial cell differentiation	0.0236552	0.082579	84.54812	1	1
GO:0045616	regulation of keratinocyte differentiation	0.0236552	0.082579	84.54812	1	1
GO:0046713	boron transport	0.0236552	0.082579	84.54812	1	1
GO:0048701	embryonic cranial skeleton morphogenesis	0.0236552	0.082579	84.54812	1	1
GO:0051771	negative regulation of nitric-oxide synthase biosynthetic process	0.0236552	0.082579	84.54812	1	1
GO:0060166	olfactory pit development	0.0236552	0.082579	84.54812	1	1
GO:0016192	vesicle-mediated transport	0.0255373	0.084476	2.601481	8	260
GO:0007420	brain development	0.0294558	0.087849	3.493724	5	121
GO:0048205	COPI coating of Golgi vesicle	0.0300247	0.088285	10.56851	2	16
GO:0006469	negative regulation of protein kinase activity	0.034276	0.091186	5.514008	3	46
GO:0008643	carbohydrate transport	0.034276	0.091186	5.514008	3	46
GO:0006629	lipid metabolic process	0.0347319	0.091465	2.099389	11	443

GO:0009615	response to virus	0.0355927	0.091976	3.328666	5	127
GO:0007155	cell adhesion	0.0358377	0.092118	1.767919	17	813
GO:0006183	GTP biosynthetic process	0.0376911	0.093146	9.394235	2	18
GO:0006228	UTP biosynthetic process	0.0376911	0.093146	9.394235	2	18
GO:0032526	response to retinoic acid	0.0376911	0.093146	9.394235	2	18
GO:0007229	integrin-mediated signaling pathway	0.038366	0.0935	3.932471	4	86
GO:0019722	calcium-mediated signaling	0.0383861	0.093511	5.284257	3	48
GO:0006461	protein complex assembly	0.0384238	0.09353	2.866038	6	177
GO:0007264	small GTPase mediated signal transduction	0.0395405	0.094095	2.257962	9	337
GO:0016337	cell-cell adhesion	0.0413694	0.094967	3.843096	4	88
GO:0006241	CTP biosynthetic process	0.0417997	0.095163	8.899802	2	19
GO:0006509	membrane protein ectodomain proteolysis	0.0417997	0.095163	8.899802	2	19
GO:0016051	carbohydrate biosynthetic process	0.0417997	0.095163	8.899802	2	19
GO:0018105	peptidyl-serine phosphorylation	0.0417997	0.095163	8.899802	2	19
GO:0055085	transmembrane transport	0.0417997	0.095163	8.899802	2	19
GO:0030163	protein catabolic process	0.0450398	0.096543	4.973419	3	51
GO:0043406	positive regulation of MAP kinase activity	0.0460855	0.096954	8.454812	2	20
GO:0050880	regulation of blood vessel size	0.0460855	0.096954	8.454812	2	20
GO:0001519	peptide amidation	0.0470311	0.097313	42.27406	1	2
GO:0001887	selenium metabolic process	0.0470311	0.097313	42.27406	1	2
GO:0002376	immune system process	0.0470311	0.097313	42.27406	1	2
GO:0006543	glutamine catabolic process	0.0470311	0.097313	42.27406	1	2
GO:0007136	meiotic prophase II	0.0470311	0.097313	42.27406	1	2
GO:0007620	copulation	0.0470311	0.097313	42.27406	1	2
GO:0009313	oligosaccharide catabolic process	0.0470311	0.097313	42.27406	1	2
GO:0009450	gamma-aminobutyric acid catabolic process	0.0470311	0.097313	42.27406	1	2
GO:0015727	lactate transport	0.0470311	0.097313	42.27406	1	2
GO:0017187	peptidyl-glutamic acid carboxylation	0.0470311	0.097313	42.27406	1	2
GO:0019049	evasion of host defenses by virus	0.0470311	0.097313	42.27406	1	2
GO:0019858	cytosine metabolic process	0.0470311	0.097313	42.27406	1	2
GO:0020027	hemoglobin metabolic process	0.0470311	0.097313	42.27406	1	2
GO:0033144	negative regulation of steroid hormone receptor signaling pathway	0.0470311	0.097313	42.27406	1	2

GO:0033280	response to vitamin D	0.0470311	0.097313	42.27406	1	2
GO:0033692	cellular polysaccharide biosynthetic process	0.0470311	0.097313	42.27406	1	2
GO:0034605	cellular response to heat	0.0470311	0.097313	42.27406	1	2
GO:0040020	regulation of meiosis	0.0470311	0.097313	42.27406	1	2
GO:0042095	interferon-gamma biosynthetic process	0.0470311	0.097313	42.27406	1	2
GO:0042363	fat-soluble vitamin catabolic process	0.0470311	0.097313	42.27406	1	2
GO:0043576	regulation of respiratory gaseous exchange	0.0470311	0.097313	42.27406	1	2
GO:0043652	engulfment of apoptotic cell	0.0470311	0.097313	42.27406	1	2
GO:0046887	positive regulation of hormone secretion	0.0470311	0.097313	42.27406	1	2
GO:0048013	ephrin receptor signaling pathway	0.0470311	0.097313	42.27406	1	2
GO:0048147	negative regulation of fibroblast proliferation	0.0470311	0.097313	42.27406	1	2
GO:0048250	mitochondrial iron ion transport	0.0470311	0.097313	42.27406	1	2
GO:0048278	vesicle docking	0.0470311	0.097313	42.27406	1	2
GO:0050790	regulation of catalytic activity	0.0470311	0.097313	42.27406	1	2
GO:0050893	sensory processing	0.0470311	0.097313	42.27406	1	2
GO:0051541	elastin metabolic process	0.0470311	0.097313	42.27406	1	2
GO:0060027	convergent extension involved in gastrulation	0.0470311	0.097313	42.27406	1	2
GO:0007596	blood coagulation	0.0475936	0.097521	3.085698	5	137
GO:0042742	defense response to bacterium	0.0475936	0.097521	3.085698	5	137
GO:0044419	interspecies interaction between organisms	0.0497488	0.09894	2.067191	10	409
GO:0016049	cell growth	0.0498017	0.098974	4.785742	3	53
**genes down-regulated**						
GO:0006955	immune response	0.0058487	0.304351	0.203976	2	829
GO:0007243	protein kinase cascade	0.0062391	0.304351	4.227406	6	120
GO:0006412	translation	0.0159007	0.304351	0.146531	1	577
GO:0009605	response to external stimulus	0.0199775	0.304351	13.0074	2	13
GO:0000398	nuclear mRNA splicing, via spliceosome	0.0217931	0.304351	2.915452	7	203
GO:0008037	cell recognition	0.023126	0.304351	12.0783	2	14
GO:0001711	endodermal cell fate commitment	0.0236552	0.304351	84.54812	1	1
GO:0007035	vacuolar acidification	0.0236552	0.304351	84.54812	1	1
GO:0014010	Schwann cell proliferation	0.0236552	0.304351	84.54812	1	1

GO:0030858	positive regulation of epithelial cell differentiation	0.0236552	0.304351	84.54812	1	1
GO:0032354	response to follicle-stimulating hormone stimulus	0.0236552	0.304351	84.54812	1	1
GO:0034695	response to prostaglandin E stimulus	0.0236552	0.304351	84.54812	1	1
GO:0034699	response to luteinizing hormone stimulus	0.0236552	0.304351	84.54812	1	1
GO:0042375	quinone cofactor metabolic process	0.0236552	0.304351	84.54812	1	1
GO:0042524	negative regulation of tyrosine phosphorylation of Stat5 protein	0.0236552	0.304351	84.54812	1	1
GO:0046108	uridine metabolic process	0.0236552	0.304351	84.54812	1	1
GO:0046164	alcohol catabolic process	0.0236552	0.304351	84.54812	1	1
GO:0046777	protein amino acid autophosphorylation	0.0253961	0.304351	4.449901	4	76
GO:0018105	peptidyl-serine phosphorylation	0.0417997	0.330225	8.899802	2	19
GO:0050896	response to stimulus	0.0421645	0.330602	0.336398	3	754
GO:0000904	cell morphogenesis involved in differentiation	0.0470311	0.335132	42.27406	1	2
GO:0006043	glucosamine catabolic process	0.0470311	0.335132	42.27406	1	2
GO:0006664	glycolipid metabolic process	0.0470311	0.335132	42.27406	1	2
GO:0018343	protein farnesylation	0.0470311	0.335132	42.27406	1	2
GO:0031574	S-M checkpoint	0.0470311	0.335132	42.27406	1	2
GO:0032331	negative regulation of chondrocyte differentiation	0.0470311	0.335132	42.27406	1	2
GO:0033595	response to genistein	0.0470311	0.335132	42.27406	1	2
GO:0033600	negative regulation of mammary gland epithelial cell proliferation	0.0470311	0.335132	42.27406	1	2
GO:0035067	negative regulation of histone acetylation	0.0470311	0.335132	42.27406	1	2
GO:0043009	chordate embryonic development	0.0470311	0.335132	42.27406	1	2
GO:0048178	negative regulation of hepatocyte growth factor biosynthetic process	0.0470311	0.335132	42.27406	1	2
GO:0050794	regulation of cellular process	0.0470311	0.335132	42.27406	1	2
GO:0050999	regulation of nitric-oxide synthase activity	0.0470311	0.335132	42.27406	1	2

Supplemental table 4.The dysregulated genes in the dysregulated pathways identified by the microarray.

path_id	path_name		gene_name	pvalue	FDR	enrichment
4142	Lysosome	UP	GM2A	2.082E-10	2.011E-08	10.292814
4142	Lysosome	UP	NAPA	2.082E-10	2.011E-08	10.292814
4142	Lysosome	UP	ASAH1	2.082E-10	2.011E-08	10.292814
4142	Lysosome	UP	GALC	2.082E-10	2.011E-08	10.292814
4142	Lysosome	UP	LIPA	2.082E-10	2.011E-08	10.292814
4142	Lysosome	UP	PSAP	2.082E-10	2.011E-08	10.292814
4142	Lysosome	UP	FUCA1	2.082E-10	2.011E-08	10.292814
4142	Lysosome	UP	LAPTM4B	2.082E-10	2.011E-08	10.292814
4142	Lysosome	UP	CTSD	2.082E-10	2.011E-08	10.292814
4142	Lysosome	UP	CTSB	2.082E-10	2.011E-08	10.292814
4142	Lysosome	UP	AP1M1	2.082E-10	2.011E-08	10.292814
4142	Lysosome	UP	IDS	2.082E-10	2.011E-08	10.292814
4142	Lysosome	UP	HGSNAT	2.082E-10	2.011E-08	10.292814
4142	Lysosome	UP	NPC1	2.082E-10	2.011E-08	10.292814
4510	Focal adhesion	UP	CAV2	0.0003225	0.0067901	4.1855504
4510	Focal adhesion	UP	PTK2	0.0003225	0.0067901	4.1855504
4510	Focal adhesion	UP	PAK2	0.0003225	0.0067901	4.1855504
4510	Focal adhesion	UP	SHC1	0.0003225	0.0067901	4.1855504
4510	Focal adhesion	UP	LAMB3	0.0003225	0.0067901	4.1855504
4510	Focal adhesion	UP	MAPK1	0.0003225	0.0067901	4.1855504
4510	Focal adhesion	UP	THBS1	0.0003225	0.0067901	4.1855504
4510	Focal adhesion	UP	CDKN1A	0.0003225	0.0067901	4.1855504
4510	Focal adhesion	UP	LAMA3	0.0003225	0.0067901	4.1855504
4510	Focal adhesion	UP	LAMA5	0.0003225	0.0067901	4.1855504
4115	p53 signaling pathway	UP	CCNG2	0.0003388	0.0069028	7.3520102
4115	p53 signaling pathway	UP	FAS	0.0003388	0.0069028	7.3520102
4115	p53 signaling pathway	UP	SERPINB5	0.0003388	0.0069028	7.3520102
4115	p53 signaling pathway	UP	SFN	0.0003388	0.0069028	7.3520102
4115	p53 signaling pathway	UP	THBS1	0.0003388	0.0069028	7.3520102
4115	p53 signaling pathway	UP	CDKN1A	0.0003388	0.0069028	7.3520102
5200	Pathways in cancer	UP	FAS	0.0003662	0.0070753	3.3206209
5200	Pathways in cancer	UP	PTK2	0.0003662	0.0070753	3.3206209
5200	Pathways in cancer	UP	TRAF4	0.0003662	0.0070753	3.3206209
5200	Pathways in cancer	UP	LAMB3	0.0003662	0.0070753	3.3206209
5200	Pathways in cancer	UP	CDKN1A	0.0003662	0.0070753	3.3206209
5200	Pathways in cancer	UP	TGFBR2	0.0003662	0.0070753	3.3206209
5200	Pathways in cancer	UP	FZD6	0.0003662	0.0070753	3.3206209
5200	Pathways in cancer	UP	SMAD3	0.0003662	0.0070753	3.3206209
5200	Pathways in cancer	UP	MAPK1	0.0003662	0.0070753	3.3206209

5200	Pathways in cancer	UP	LAMA3	0.0003662	0.0070753	3.3206209
5200	Pathways in cancer	UP	LAMA5	0.0003662	0.0070753	3.3206209
5200	Pathways in cancer	UP	IKBKB	0.0003662	0.0070753	3.3206209
5200	Pathways in cancer	UP	SLC2A1	0.0003662	0.0070753	3.3206209
5220	Chronic myeloid leukemia	UP	SHC1	0.0005362	0.0086344	6.7638494
5220	Chronic myeloid leukemia	UP	TGFBR2	0.0005362	0.0086344	6.7638494
5220	Chronic myeloid leukemia	UP	CDKN1A	0.0005362	0.0086344	6.7638494
5220	Chronic myeloid leukemia	UP	SMAD3	0.0005362	0.0086344	6.7638494
5220	Chronic myeloid leukemia	UP	MAPK1	0.0005362	0.0086344	6.7638494
5220	Chronic myeloid leukemia	UP	IKBKB	0.0005362	0.0086344	6.7638494
250	Alanine, aspartate and glutamate metabolism	UP	ABAT	0.001069	0.0132123	10.568515
250	Alanine, aspartate and glutamate metabolism	UP	ADSSL1	0.001069	0.0132123	10.568515
250	Alanine, aspartate and glutamate metabolism	UP	GLS	0.001069	0.0132123	10.568515
250	Alanine, aspartate and glutamate metabolism	UP	GFPT1	0.001069	0.0132123	10.568515
5222	Small cell lung cancer	UP	IKBKB	0.0011228	0.013559	5.8987058
5222	Small cell lung cancer	UP	PTK2	0.0011228	0.013559	5.8987058
5222	Small cell lung cancer	UP	TRAF4	0.0011228	0.013559	5.8987058
5222	Small cell lung cancer	UP	LAMB3	0.0011228	0.013559	5.8987058
5222	Small cell lung cancer	UP	LAMA3	0.0011228	0.013559	5.8987058
5222	Small cell lung cancer	UP	LAMA5	0.0011228	0.013559	5.8987058
4360	Axon guidance	UP	PTK2	0.001795	0.0192685	4.5878823
4360	Axon guidance	UP	PAK2	0.001795	0.0192685	4.5878823
4360	Axon guidance	UP	RHOD	0.001795	0.0192685	4.5878823
4360	Axon guidance	UP	MAPK1	0.001795	0.0192685	4.5878823
4360	Axon guidance	UP	CDKN1A	0.001795	0.0192685	4.5878823
4360	Axon guidance	UP	EPHA2	0.001795	0.0192685	4.5878823
4360	Axon guidance	UP	PLXNB2	0.001795	0.0192685	4.5878823
4512	ECM-receptor interaction	UP	CD44	0.0065072	0.0540683	5.032626
4512	ECM-receptor interaction	UP	LAMB3	0.0065072	0.0540683	5.032626
4512	ECM-receptor interaction	UP	THBS1	0.0065072	0.0540683	5.032626
4512	ECM-receptor interaction	UP	LAMA3	0.0065072	0.0540683	5.032626
4512	ECM-receptor interaction	UP	LAMA5	0.0065072	0.0540683	5.032626
4012	ErbB signaling pathway	UP	PTK2	0.0075676	0.0598371	4.8590872
4012	ErbB signaling pathway	UP	PAK2	0.0075676	0.0598371	4.8590872
4012	ErbB signaling pathway	UP	SHC1	0.0075676	0.0598371	4.8590872
4012	ErbB signaling pathway	UP	MAPK1	0.0075676	0.0598371	4.8590872
4012	ErbB signaling pathway	UP	CDKN1A	0.0075676	0.0598371	4.8590872
4350	TGF-beta signaling pathway	UP	TFDP1	0.0075676	0.0598371	4.8590872
4350	TGF-beta signaling pathway	UP	MAPK1	0.0075676	0.0598371	4.8590872
4350	TGF-beta signaling pathway	UP	THBS1	0.0075676	0.0598371	4.8590872

4350	TGF-beta signaling pathway	UP	TGFBR2	0.0075676	0.0598371	4.8590872
4350	TGF-beta signaling pathway	UP	SMAD3	0.0075676	0.0598371	4.8590872
4650	Natural killer cell mediated cytotoxicity	UP	FAS	0.0118843	0.0785058	3.7028372
4650	Natural killer cell mediated cytotoxicity	UP	TNFRSF10D	0.0118843	0.0785058	3.7028372
4650	Natural killer cell mediated cytotoxicity	UP	SHC1	0.0118843	0.0785058	3.7028372
4650	Natural killer cell mediated cytotoxicity	UP	IFNGR1	0.0118843	0.0785058	3.7028372
4650	Natural killer cell mediated cytotoxicity	UP	MAPK1	0.0118843	0.0785058	3.7028372
4650	Natural killer cell mediated cytotoxicity	UP	MICA	0.0118843	0.0785058	3.7028372
4910	Insulin signaling pathway	UP	PHKB	0.0123036	0.0799972	3.6760051
4910	Insulin signaling pathway	UP	FAS	0.0123036	0.0799972	3.6760051
4910	Insulin signaling pathway	UP	SHC1	0.0123036	0.0799972	3.6760051
4910	Insulin signaling pathway	UP	EXOC7	0.0123036	0.0799972	3.6760051
4910	Insulin signaling pathway	UP	MAPK1	0.0123036	0.0799972	3.6760051
4910	Insulin signaling pathway	UP	IKBKB	0.0123036	0.0799972	3.6760051
10	Glycolysis / Gluconeogenesis	UP	PGM1	0.0125572	0.0808765	5.4547172
10	Glycolysis / Gluconeogenesis	UP	ALDH1A3	0.0125572	0.0808765	5.4547172
10	Glycolysis / Gluconeogenesis	UP	PKM2	0.0125572	0.0808765	5.4547172
10	Glycolysis / Gluconeogenesis	UP	ADH5	0.0125572	0.0808765	5.4547172
5211	Renal cell carcinoma	UP	PAK2	0.0191758	0.1075182	4.831321
5211	Renal cell carcinoma	UP	CDKN1A	0.0191758	0.1075182	4.831321
5211	Renal cell carcinoma	UP	MAPK1	0.0191758	0.1075182	4.831321
5211	Renal cell carcinoma	UP	SLC2A1	0.0191758	0.1075182	4.831321
5212	Pancreatic cancer	UP	TGFBR2	0.0211248	0.1140971	4.6971176
5212	Pancreatic cancer	UP	SMAD3	0.0211248	0.1140971	4.6971176
5212	Pancreatic cancer	UP	MAPK1	0.0211248	0.1140971	4.6971176
5212	Pancreatic cancer	UP	IKBKB	0.0211248	0.1140971	4.6971176
4520	Adherens junction	UP	CTNND1	0.0265436	0.1300853	4.39211
4520	Adherens junction	UP	TGFBR2	0.0265436	0.1300853	4.39211
4520	Adherens junction	UP	MAPK1	0.0265436	0.1300853	4.39211
4520	Adherens junction	UP	SMAD3	0.0265436	0.1300853	4.39211
5219	Bladder cancer	UP	THBS1	0.0268329	0.1308561	6.0391512
5219	Bladder cancer	UP	CDKN1A	0.0268329	0.1308561	6.0391512
5219	Bladder cancer	UP	MAPK1	0.0268329	0.1308561	6.0391512
520	Amino sugar and	UP	PGM1	0.028597	0.1353963	5.8987058

	nucleotide sugar metabolism					
520	Amino sugar and nucleotide sugar metabolism	UP	PMM2	0.028597	0.1353963	5.8987058
520	Amino sugar and nucleotide sugar metabolism	UP	GFPT1	0.028597	0.1353963	5.8987058
100	Steroid biosynthesis	UP	SC4MOL	0.0337644	0.1472928	9.9468373
100	Steroid biosynthesis	UP	LIPA	0.0337644	0.1472928	9.9468373
5210	Colorectal cancer	UP	TGFBR2	0.0355015	0.1508826	4.0261008
5210	Colorectal cancer	UP	FZD6	0.0355015	0.1508826	4.0261008
5210	Colorectal cancer	UP	SMAD3	0.0355015	0.1508826	4.0261008
5210	Colorectal cancer	UP	MAPK1	0.0355015	0.1508826	4.0261008
4930	Type II diabetes mellitus	UP	PKM2	0.0362985	0.1524686	5.3966883
4930	Type II diabetes mellitus	UP	MAPK1	0.0362985	0.1524686	5.3966883
4930	Type II diabetes mellitus	UP	IKBKB	0.0362985	0.1524686	5.3966883
531	Glycosaminoglycan degradation	UP	HGSNAT	0.0417997	0.1682604	8.8998018
531	Glycosaminoglycan degradation	UP	IDS	0.0417997	0.1682604	8.8998018
4520	Adherens junction	DOWN	FGFR1	0.000619	0.0547099	6.588165
4520	Adherens junction	DOWN	NLK	0.000619	0.0547099	6.588165
4520	Adherens junction	DOWN	ACTB	0.000619	0.0547099	6.588165
4520	Adherens junction	DOWN	CTNNB1	0.000619	0.0547099	6.588165
4520	Adherens junction	DOWN	RHOA	0.000619	0.0547099	6.588165
4520	Adherens junction	DOWN	EGFR	0.000619	0.0547099	6.588165
5130	Pathogenic Escherichia coli infection - EHEC	DOWN	TUBA3C	0.0008153	0.0708265	7.9762375
5130	Pathogenic Escherichia coli infection - EHEC	DOWN	TUBB2A	0.0008153	0.0708265	7.9762375
5130	Pathogenic Escherichia coli infection - EHEC	DOWN	ACTB	0.0008153	0.0708265	7.9762375
5130	Pathogenic Escherichia coli infection - EHEC	DOWN	CTNNB1	0.0008153	0.0708265	7.9762375
5130	Pathogenic Escherichia coli infection - EHEC	DOWN	RHOA	0.0008153	0.0708265	7.9762375
4530	Tight junction	DOWN	VAPA	0.0106887	0.4998421	3.7857366
4530	Tight junction	DOWN	ACTB	0.0106887	0.4998421	3.7857366
4530	Tight junction	DOWN	CASK	0.0106887	0.4998421	3.7857366

4520	Adherens junction	DOWN	FGFR1	0.000619	0.0547099	6.588165
4520	Adherens junction	DOWN	NLK	0.000619	0.0547099	6.588165
4520	Adherens junction	DOWN	ACTB	0.000619	0.0547099	6.588165
4520	Adherens junction	DOWN	CTNNB1	0.000619	0.0547099	6.588165
4520	Adherens junction	DOWN	RHOA	0.000619	0.0547099	6.588165
4520	Adherens junction	DOWN	EGFR	0.000619	0.0547099	6.588165

5130	Pathogenic Escherichia coli infection - EHEC	DOWN	TUBA3C	0.0008153	0.0708265	7.9762375
5130	Pathogenic Escherichia coli infection - EHEC	DOWN	TUBB2A	0.0008153	0.0708265	7.9762375
5130	Pathogenic Escherichia coli infection - EHEC	DOWN	ACTB	0.0008153	0.0708265	7.9762375
5130	Pathogenic Escherichia coli infection - EHEC	DOWN	CTNNB1	0.0008153	0.0708265	7.9762375
5130	Pathogenic Escherichia coli infection - EHEC	DOWN	RHOA	0.0008153	0.0708265	7.9762375
4530	Tight junction	DOWN	VAPA	0.0106887	0.4998421	3.7857366
4530	Tight junction	DOWN	ACTB	0.0106887	0.4998421	3.7857366
4530	Tight junction	DOWN	CASK	0.0106887	0.4998421	3.7857366
4530	Tight junction	DOWN	MYH10	0.0106887	0.4998421	3.7857366
4530	Tight junction	DOWN	CTNNB1	0.0106887	0.4998421	3.7857366
4530	Tight junction	DOWN	RHOA	0.0106887	0.4998421	3.7857366

Supplemental table 5 The basic clinic parameters of patients enrolled in this study

Case number	ID of inpatient	Gender	Age(years)	Date of surgery	Diagnosis of histopathology	Tumor size	Distant metastasis	T	N	M	AJCC clinical stage	History of diabetes
505	1005292078	Male	53	10-May-16	PDAC	1.5×1×1cm	No	T3	N0	M0	2A	YES
no	1005122842	Male	61	17-May-16	PDAC	9×6×6cm	No	T3	N0	M1	4	YES
524	1005566398	Male	49	10-Jun-16	PDAC	5×3×2cm	No	T2	N1	M0	2B	unknown
534	1005475368	Female	64	8-Jul-16	PDAC	4×4×2cm	No	T2	N0	M0	1B	YES
538	1001614445	Female	74	19-Jul-16	PDAC	8×5×3cm	No	T3	N0	M0	2A	unknown
548	1005759755	Female	64	8-Aug-16	PDAC	10×5×4cm	No	T2	N0	M0	1B	YES
653	1004908044	Male	60	13-Mar-17	PDAC	3×3×2.5cm	No	T2	N1	M0	2B	YES
654	1006147126	Male	38	14-Mar-17	PDAC	6.5×4×3.5cm	No	T2	N1	M0	2B	unknown
696	1006554313	Male	49	13-Jun-17	PDAC	7×5×3cm	No	T3		M0	2	YES
706	1006872878	Female	75	25-Jul-17	PDAC	4×4×3cm	No	T3	N0	M1	4	unknown
727	1006763952	Male	73	29-Sep-17	PDAC	7×4×3cm	No	T2		M0	2A	YES
